# The Simons Genome Diversity Project: A Global Analysis of Mobile Element Diversity

**DOI:** 10.1093/gbe/evaa086

**Published:** 2020-05-02

**Authors:** W Scott Watkins, Julie E Feusier, Jainy Thomas, Clement Goubert, Swapon Mallick, Lynn B Jorde

**Affiliations:** e1 Department of Human Genetics, University of Utah; e2 Department of Molecular Biology and Genetics, Cornell University; e3 Department of Genetics, Harvard Medical School, Boston, Massachusetts

**Keywords:** mobile elements, Simons Genome Diversity Project, genetic diversity, population genetics, Native Americans, retrotransposition

## Abstract

Ongoing retrotransposition of *Alu*, LINE-1, and SINE–VNTR–*Alu* elements generates diversity and variation among human populations. Previous analyses investigating the population genetics of mobile element insertions (MEIs) have been limited by population ascertainment bias or by relatively small numbers of populations and low sequencing coverage. Here, we use 296 individuals representing 142 global populations from the Simons Genome Diversity Project (SGDP) to discover and characterize MEI diversity from deeply sequenced whole-genome data. We report 5,742 MEIs not originally reported by the 1000 Genomes Project and show that high sampling diversity leads to a 4- to 7-fold increase in MEI discovery rates over the original 1000 Genomes Project data. As a result of negative selection, nonreference polymorphic MEIs are underrepresented within genes, and MEIs within genes are often found in the transcriptional orientation opposite that of the gene. Globally, 80% of *Alu* subfamilies predate the expansion of modern humans from Africa. Polymorphic MEIs show heterozygosity gradients that decrease from Africa to Eurasia to the Americas, and the number of MEIs found uniquely in a single individual are also distributed in this general pattern. The maximum fraction of MEI diversity partitioned among the seven major SGDP population groups (*F*_ST_) is 7.4%, similar to, but slightly lower than, previous estimates and likely attributable to the diverse sampling strategy of the SGDP. Finally, we utilize these MEIs to extrapolate the primary Native American shared ancestry component to back to Asia and provide new evidence from genome-wide identical-by-descent genetic markers that add additional support for a southeastern Siberian origin for most Native Americans.

## Introduction

In humans, a single class of transposable elements, the retrotransposons, accounts for nearly all current mobile DNA activity (Beck et al. 2011; Kazazian and Moran 2017). Through ∼65 Myr of prolific activity in primate lineages, retrotransposons now constitute at least 42% of the human genome (Lander et al. 2001; Batzer and Deininger 2002; Kazazian and Moran 2017). Most mobile element insertions (MEIs) are static entities, often inactivated by mutation, methylation, and piRNAs, and incapable of further transposition. Yet, each person harbors ∼80–100 full-length potentially active long interspersed elements (LINE-1; Brouha et al. 2003) that can autonomously retrotranspose and mobilize other nonautonomous short interspersed elements (SINEs), such as *Alu* and SINE–VNTR–*Alu* (SVA) elements.

Collectively, mobile element activity creates an ongoing evolutionary process that influences genome structure and gene function (Beck et al. 2010; Richardson et al. 2015). For example, new germline *Alu*, LINE-1, and SVA insertions can give rise to inherited disorders (e.g., neurofibromatosis 1) (Wimmer et al. 2011) and contribute to a predisposition to some cancer types (e.g., prostate/ovarian, head/neck, and gastrointestinal cancers) (Wimmer et al. 2011; Burns 2017). New somatic LINE-1 insertions can inactivate key tumor suppressors, such as the *APC* gene, and lead directly to colon cancer phenotypes (Scott et al. 2016). MEI-mediated nonallelic recombination may also drive disease processes by altering gene function and gene expression (e.g., *BRCA1*, Su et al. 2018; *FCMD*, Taniguchi-Ikeda et al. 2011). Although MEI activity can cause genetic disease, the bulk of retrotransposition is initially neutral (Cordaux et al. 2006) and occurs at a regular rate. Based on an analysis of 599 individuals from 33 nondisease pedigrees, the minimum germline retrotransposition rates in humans are 0.016 LINE-1s per birth, 0.025 *Alus* per birth, and 0.016 SVAs per birth or 1 in ~40–63 births overall (Feusier et al. 2019).

Retrotransposition produces de novo insertions that are mostly unbiased with respect to genomic location, constrained only to a limited degree by the loose sequence specificity of the LINE-1 encoded endonuclease/reverse transcriptase that catalyzes retrotransposition (Flasch et al. 2019; Sultana et al. 2019). This process gives rise to insertion/deletion polymorphisms that are identical by decent, have known ancestral and derived states, and are essentially homoplasy free (Batzer and Deininger 2002; Doronina et al. 2019). These properties make retrotransposons ideal genetic markers for evolutionary questions, and they have been used across primates to accurately infer phylogenies within and among species (Hormozdiari et al. 2013; Steely et al. 2018).

Early studies using a small number of loci demonstrate that MEIs accurately resolve evolutionary relationships among world population groups (Watkins et al. 2003; Witherspoon et al. 2006). These studies, however, were limited by low numbers of markers and various biases in marker ascertainment. Whole-genome sequencing (WGS) and recent improvements in algorithms for MEI discovery and genotyping are now giving rise to MEI data sets with fewer biases, primarily, because the polymorphic MEI discovery process occurs in all individuals from all populations. One such study, the 1000 Genomes Project, identified more than 16,600 polymorphic MEIs (Sudmant et al. 2015; Gardner et al. 2017) and has provided a framework for MEI-based relationships among 26 world populations (Rishishwar et al. 2015). Yet, despite a relatively large number of samples, the 1000 Genomes Project is limited by the relatively small number of populations and sampling locations. The growing availability of large WGS data sets can further facilitate the analysis of population variation, including variation in mobile elements, in comprehensive detail (UK10K Consortium et al. 2015; Pagani et al. 2016; Rishishwar et al. 2017; Feusier et al. 2019; Puurand et al. 2019).

In this study, we leverage the genomic diversity of the Simons Genome Diversity Project (SGDP) (Mallick et al. 2016) to investigate the global population dynamics of polymorphic *Alu*, LINE-1, and SVA elements. These data are drawn from 296 deeply sequenced (43× average depth) whole genomes from 142 populations in 75 well-defined geographic regions of the Old and New World and include many populations never before assayed for mobile element diversity. Here, we present thousands of new MEIs, quantify the genetic relationships among populations and individuals using MEI and comparative single nucleotide polymorphism (SNP) data, examine population sampling strategies for estimating diversity, use MEI-based ancestry estimates to analyze the origins of New World population in Asia, and quantify *Alu* subfamily activity in the SGDP populations.

## Results and Discussion

### MEI Discovery in the SGDP Samples

**Fig. 1.**
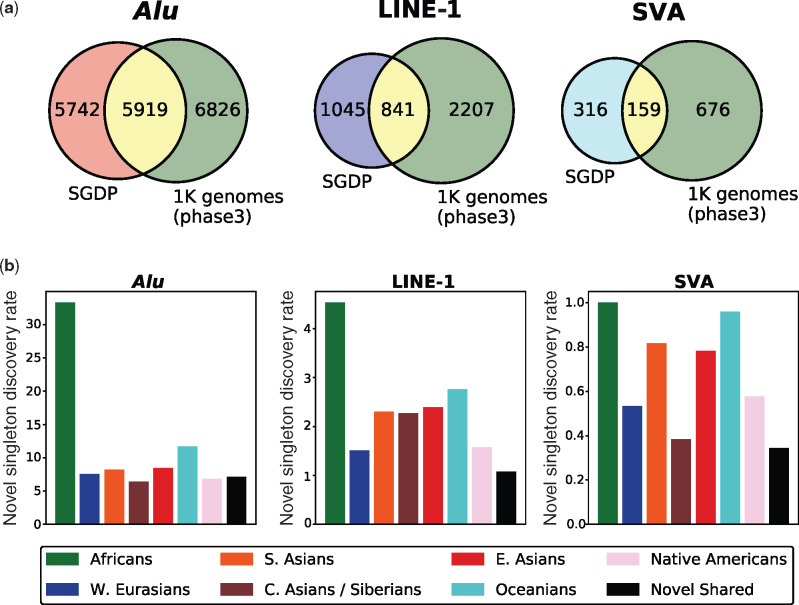
—Comparison of the SGDP MEI loci to the 1000 Genomes Project (phase 3) MEI loci. (*a*) Venn diagrams show the intersection of nonreference MEIs discovered in the SGDP (296 individuals) and the 1000 Genomes Project (phase 3) data sets (2,504 individuals). The per sample MEI discovery rates were ∼4- to 7-fold higher in the SGDP than the 1000 genomes collection. Discovery rates were highest for the nonreference *Alu* insertions. A window of ±25 bp was used to determine positional overlap between the data sets. (*b*) A bar plot shows the normalized rate of novel singleton MEIs discovery per individual in the SGDP for each major population group. *Alu* singleton insertions were discovered at a higher rate than LINE-1 or SVA singleton insertions. Africans consistently had the highest rate of discovery. The black bar shows the discovery rate of novel nonsingleton MEIs. Rates are calculated as the number of novel MEIs counts divided by the number of individuals in the population (Africans: 49, West Eurasians: 75, South Asians: 49, Central Asians and Siberians: 26, East Asians: 46, Oceanians: 25, Native Americans: 26, and all populations: 296).

*Alu*, LINE-1, and SVA retrotransposons were identified and genotyped in 296 individuals from 75 geopolitical regions represented in the SGDP. (supplementary tables 1 and 2, Supplementary Material online). We discovered 11,661 *Alu*; 1,886 LINE-1; and 475 SVA polymorphic MEIs not present in the human reference genome (build 38) using the mobile element locator tool (MELT v2.1.4) (Gardner et al. 2017). Approximately half of the MEIs (5,742 *Alus*; 1,045 LINE-1s; and 316 SVAs) are novel with respect to those originally reported by the 1000 Genomes Project (Sudmant et al. 2015) (fig. 1*a* and supplementary fig. 1, Supplementary Material online). For MEI loci found in both data sets, the MEI frequencies, calculated over all populations, were similar between studies (*r* ≥ 0.98, Pearson correlation). Additionally, there was generally good agreement between nonreference genotypes obtained computationally that passed Hardy–Weinberg equilibrium (HWE) filtering and those obtained by polymerase chain reaction (94% concordance, five loci). The MELT software also genotypes MEI insertions present in the reference sequence but with a concordance rate of ∼70% compared with polymerase chain reaction-based data (Goubert et al. 2020). We use nonreference MEI insertions for all analyses except where specifically noted.

Despite a comparatively low number of individuals in the SGDP (296 individuals from 142 populations), the rate of new retrotransposon discovery was substantially higher in the SGDP than in the 1000 Genomes Project (2,504 individuals from 26 populations; Rishishwar et al. 2015; Sudmant et al. 2015). The 1000 Genomes Project found 16,631 MEIs in 2,504 individuals for an average discovery rate of 6.6 MEIs per individual. In contrast, the total nonreference MEI discovery rate for the SGDP was ∼7-fold higher: 14,022 MEIs/296 individuals = 47.4 MEIs per individual. The novel MEI discovery rate was also high in the SGDP data (fig. 1*b*). For instance, novel singleton *Alu* elements were found at a rate of ∼33 insertions per individual in the African samples, demonstrating that substantial new MEI variation can be discovered by sampling new populations.

Overall, the high rate of MEI discovery in the SGDP was driven primarily by the diverse sampling of Africans. For instance, MEIs unique to the 22 distinct African populations account for 43%, 33%, and 33% of all *Alu*, LINE-1, and SVA elements, yet Africans represent only ∼17% of all SGDP samples. In contrast, New World populations contribute 290 New World-specific MEIs (∼2% of all elements) but account for 8.8% of all samples. Although the SGDP and 1000 Genomes Project used different genome builds and were sequenced to different depths, the results demonstrate that sampling a small number of individuals from many different populations maximizes discovery rates and more accurately assesses MEI diversity than sampling larger numbers of individuals from fewer populations.

Our discovery rate estimates and other population level assessments of MEIs are based on short-read sequencing data. Recent work has demonstrated that long-read sequencing data allow detection of substantially more LINE-1 insertions than short-read data, especially when new MEIs insert within existing MEIs (Zhou et al. 2019). We also note that Gardner et al. (2017) reanalyzed the 1000 Genomes Project data and found 6,089 additional MEIs. However, in the reanalysis, only 11,363 total *Alu*, LINE-1, and SVA elements had the highest MELT quality scores (ASSESS = 5; see Gardner et al. 2017, supplementary table S2), which was the minimum quality score required for inclusion in this study. Therefore, our discovery rate estimates for nonreference MEIs, based on the comparison with all 16,628 MEIs from the original 1000 genomes study shown in figure 1, are conservative. Moreover, a comparison of all (MELT PASS) calls from all quality tranches from the reanalyzed 1000 genomes data and the SGDP data produced discovery rate estimates similar in magnitude to the estimates shown above: 22,723 MEIs/2,534 individuals = 9 MEIs/individual versus 16,288 MEIs/296 individuals = 55 MEIs/individual, respectively.

The molecular features of computationally identified MEIs in the SGDP, such as target site duplication sizes and insert length distributions, are in accord with retrotransposon characteristics from previous WGS data sets and those using traditional methods to identify MEIs (Stewart et al. 2011). Eighty-three percent of *Alu* insertions and 39% of LINE-1 insertions were ≥95% of full length relative to their respective consensus sequences. The length distribution of nonreference LINE-1 elements was similar to those in previous reports (Boissinot et al. 2000, 2004). The median target site duplication size for all three MEI classes was 14–15 bp (range 0–26 bp) (supplementary fig. 2, Supplementary Material online), which likely reflects the common insertional mechanism that mediates *Alu*, LINE-1, and SVA retrotransposition (Moran et al. 1996; Raiz et al. 2012).

Although de novo LINE-1 elements may insert into most genomic locations with only limited constraint (Flasch et al. 2019; Sultana et al. 2019), the newly discovered nonreference *Alu* and LINE-1 insertions showed significant deviation from a random distribution with respect to genic insertion. Nonreference *Alu* and LINE-1 insertions were significantly underrepresented in genes (5′-UTRs, coding regions, 3′-UTR) and introns and significantly overrepresented in nongenic regions (*P* ≤ 1E−5, Fisher’s test; table 1). Ten new *Alu* insertions were found within the coding regions of ten genes (supplementary table 3 and supplementary fig. 3, Supplementary Material online). These coding insertions are expected to create a frameshift shortly beyond the insertion point and were found at very low frequency, primarily as singletons (6) or doubletons (2). The potentially deleterious insertions were found in five of the seven major population groups and found at similar frequencies in populations within and outside of Africa.

**Table 1:** MEI Locations and Gene-Related Genomic Features

Region	Size (Mb)	Observed	Expected	*P* value*
*Alu*				
3′-UTR	90.75	83	325	1E−11*
5′-UTR	21.02	15	75	1E−05*
Coding	41.94	12	150	1E−17*
Intronic	1,830.01	4,980	6,551	1E−15*
Nongenic	1,273.63	6,571	4,559	1E−23*
Total	3,257.35	11,661	11,661	–
LINE-1				
3′-UTR	90.75	13	53	1E−07*
5′-UTR	21.02	2	12	1E−11*
Coding	41.94	0	24	1E−07*
Intronic	1,830.01	725	1,060	1E−12*
Nongenic	1,273.63	1,146	737	1E−44*
Total	3,257.35	1,886	1,886	–
SVA				
3′-UTR	90.75	5	13	0.0271
5′-UTR	21.02	2	3	0.8500
Coding	41.94	1	6	0.0425
Intronic	1,830.01	216	267	0.0154
Nongenic	1,273.63	251	186	0.0006*
Total	3,257.35	475	475	–

* Significant at (Bonferroni-corrected) *P* < 0.05, Fisher’s exact test.

Considering all nonreference insertions in all genomic locations, MEIs did not differ significantly in their insertional orientation, but dividing the genome into genic and nongenic regions revealed interesting trends and biologically significant patterns. The *Alu* and LINE-1 elements inserted in exonic regions of genes, including 5′- and 3′-UTRs, were found more often in the opposite orientation of the gene’s transcription (61/110 and 9/15, respectively, not significant). There were also significantly more *Alu* and SVA elements inserted in an opposite transcriptional orientation in transcribed regions (including all introns) of genes than expected by chance (*P* ≤ 0.0001 and *P* ≤ 0.0003, proportion test). This finding suggests that there is negative selection on intragenic *Alu* and SVA elements that insert in the same transcriptional orientation of a gene even when the insertions are intronic. Additionally, there were significantly more nongenic than genic insertions for all MEI classes than expected by chance.

These results are generally in accord with previous reports of purifying selection on MEI events that occur within certain intronic regions of genes (Zhang et al. 2011). Purifying selection on MEIs and copy number variants within genes has been reported in the 1000 Genomes Project (Sudmant et al. 2015), and recent work has demonstrated that altered splicing is a possible consequence of intragenic MEI insertions (Payer et al. 2019). Other studies have also found MEIs to be inserted more frequently in the reverse orientation with respect to the transcriptional orientation of the gene, with distance to the nearest exon a factor influencing the selection bias (Medstrand et al. 2002; Zhang et al. 2011). Our results are based only on nonreference polymorphic MEIs discovered by short-read sequencing analysis, and it is possible that additional MEIs uncovered by long-read sequencing may attenuate the observed findings. Yet, using a diverse sampling of humans, our results demonstrate purifying selection against MEI insertions within genes and suggest future studies to address whether functional constraint on young polymorphic MEIs vary by gene class.

### MEI Activity and Transduction

Transcriptional read through of an active LINE-1 element can move host genomic material to new locations during retrotransposition in a process known as transduction. The transduced host material can be used to identify the active source LINE-1 element. We found ten LINE-1 transduction events originating from six LINE-1 source elements (table 2). Transduction events in the SGDP differed from those identified in the 1000 Genomes Project (Gardner et al. 2017), and “hot” LINE-1 insertions in the 1000 Genomes Project were not active transducers in these populations. The SGDP transduction events mobilized a total of 3.29 kb of intronic and intragenic sequence. Along with the LINE-1 elements, existing host sequences were transduced into the introns of six genes and four intragenic regions. The newly inserted sequences in genes were not near splice junctions. Further testing is necessary to determine if these transduction events alter gene expression or splicing patterns. The most active source element (3/10 events) was found at higher frequency (0.84) in oceanic populations than in other groups (range 0.53–0.67). The overall rate of discovery of LINE-1 elements with transduction events was 1 per 29.6 genomes, and this value was similar to 1 per 20.7 genomes observed in the 1000 Genomes Phase3 data (Gardner et al. 2017). This transduction rate estimate, however, is only a weak proxy the actual L1 retrotransposition rate because of limitations on inferring source elements and possible selection and acquisition bias against some full-length elements (Macfarlane et al. 2013).

**Table 2:** LINE-1 Transduction Events

Insertion Position	Insertion Location	Insertion Feature	Source LINE-1	Length (bp)	Source Location	Source Reference/Features
2:133163757	Intronic	*NCKAP5*	3:173031344	356	Intronic	NonRef/*SPATA16*
3:58744797	Intronic	*C3orf57*	6:102398219	72	Intergenic	NonRef
3:184969743	Intronic	*VPS8*	6:102398219	72	Intergenic	NonRef
5:163806214	Intergenic	–	4:111707817	49	Intergenic	NonRef
5:143507197	Intergenic	–	4:19083929	698	Intergenic	Ref
9:3946911	Intronic	*GLISS*	4:19083929	698	Intergenic	Ref
14:48654345	Intergenic	–	4:19083929	685	Intergenic	Ref
6:73031902	Intronic	*KCNQ5*	4:146304144	114	Intronic	NonRef/*SLC10A7*
13:60298249	Intergenic	–	6:24817706	212	Intronic	Ref/*RIPOR2*
15:99892071	Intergenic	*LINE2*	6:24817706	334	Intronic	Ref/*RIPOR2*

Note.—Ref/NonRef, present/absent in reference sequence (build 38).

### Population Structure

**Fig. 2.**
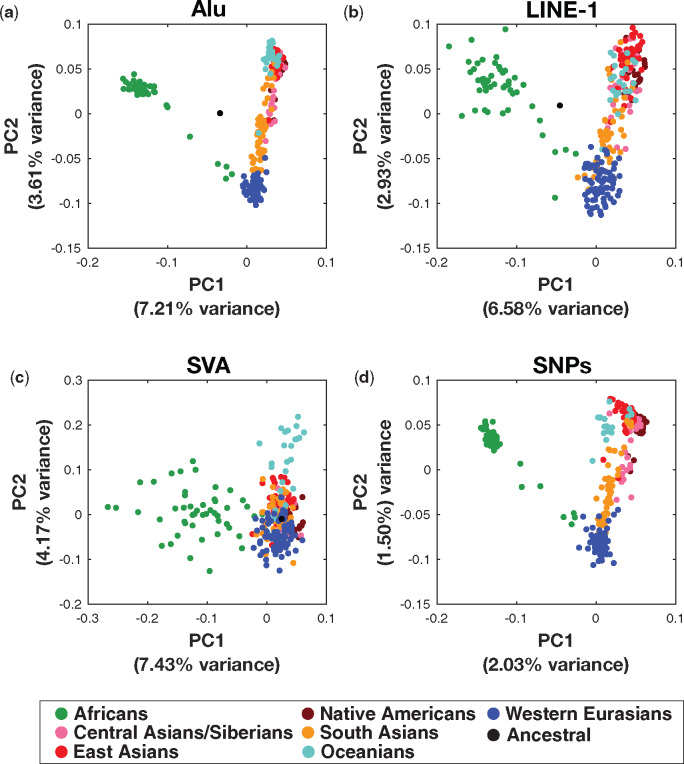
—Genetic relationships among individuals estimated from *Alu*, LINE-1, and SVA mobile element insertion polymorphisms. PCA was used to visualize the genetic distance estimates among 296 individuals from world populations. Points are color-coded by each of the seven major population groups in the SGDP. (*a*) Nonreference *Alu* markers (*n* = 2,561) separate Africans and non-Africans along PC1 and non-Africans populations are distributed along PC2. A hypothetical ancestral individual lacking *Alu* elements at all loci is located centrally. (*b*) Nonreference LINE-1 insertions (*n* = 310) produce genetic distance estimates similar to *Alu* elements, but there is substantially greater dispersion among Africans. (*c*) Genetic relationships based on SVA insertions (*n* = 96) are less structured than for *Alus* and LINE-1s, which may be attributable to a small number (65) of common SVA markers. (*d*) The genetic relationships among individuals in the SGDP based on SNPs (*n *= 347,532; CpG sites excluded) are highly congruent with *Alu* and LINE-1 polymorphic insertions. All sites used in each analysis are unlinked (*r*^2^ ≤ 0.2), have an overall minor allele frequency of ≥0.02, and have <10% missing data by sample and locus.

We compared the population structure and interindividual genetic relationships based on newly discovered nonreference MEIs to those based on SNPs. Because the MEIs were discovered using WGS data from many populations, ascertainment bias is expected to be minimal. Biases due to the unequal ethnic composition of the reference sequence may exist. For example, 70.28% of the current human genome reference (build 38) is based on cloned sequences from a single African-European individual (Reich et al. 2009; Schneider et al. 2017), which limits the diversity represented in the reference sequence (Sherman et al. 2019) and produces potential biases in all methods that rely on this resource. Interindividual genetic distances were inferred using allele-sharing distances and then visualized by principal component analysis (PCA). The genetic relationships among individuals obtained from common unlinked *Alu* (2,561) and LINE-1 (310) MEIs were highly concordant with those based on 347,532 unlinked, non-CpG, SNPs (fig. 2). Genetic distances from SVA elements showed lower concordance with the SNP-based genetic distances, but this result is likely due to a very small number (65) of common SVA elements available for analysis. Sub-Saharan Africans are notably distinct from northern Africans and other world populations along principal component (PC) 1. Western Eurasians, southern Asians, and eastern Asians are distributed according to geographic location along PC 2. Oceanians and Native Americans are less well resolved and cluster primarily with other Asians.

Assembling individuals into seven major groups yields population-based neighbor-joining genetic distance networks that reveal additional details regarding population affinities. These networks indicate that Native Americans have higher affinity to central Asians than to other populations. Oceanians are notably distinct from other Asian populations (supplementary fig. 4, Supplementary Material online).

### Genetic Diversity

**Fig. 3.**
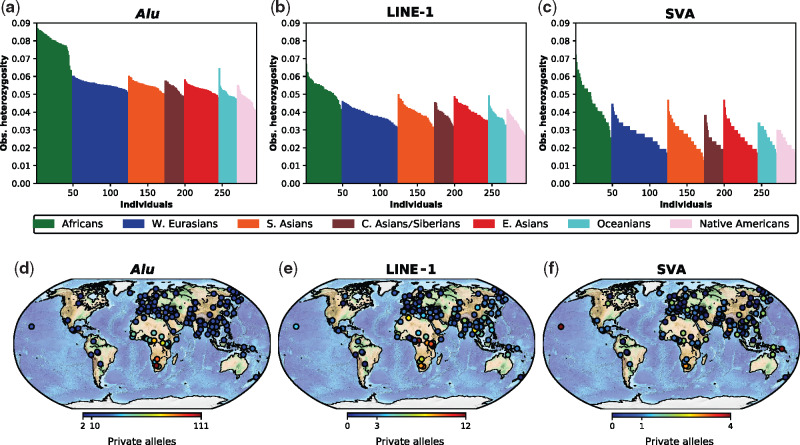
—Geographic structure of MEI heterozygosity and private alleles. (*a*–*c*) Individual MEI heterozygosity for *Alu*, LINE-1, and SVA elements are shown. Each bar represents one individual, and the height of the bar shows the observed fraction of heterozygous sites found in that sample. Samples are sorted from high to low within each of the seven major population groups. (*d*–*f*) Heat maps show the geographic location and number of private insertions for all individuals for each MEI type. (*a*–*f*) A trend of decreasing heterozygosity and decreasing private alleles with increasing distance from Africa occurs for all retrotransposon types.

To obtain a global estimate of MEI diversity, individual heterozygosity and the number of private MEI alleles per individual were evaluated with respect to geography (fig. 3). Individual MEI diversity (heterozygosity) for *Alu*, LINE-1, and SVA polymorphisms is typically higher in sub-Saharan Africans (fig. 3*a*–*c*). *Alu* elements show a relatively sharp decline in diversity for populations outside of Africa. This trend was less pronounced for LINE-1 and SVA elements. Africans generally have more MEIs found uniquely in single individuals (singletons and homozygous doubletons) (fig. 3*d*–*f*). Individual heterozygosity and the number of private alleles for MEIs are lower in New World populations than Old World populations, a finding consistent with a very significant population bottleneck and a limited number of founders for New World populations (Wall et al. 2011).

Genetic diversity trends among the seven major population groups reflect diversity among individuals. Population diversity was highest in Africans. South Asians had higher diversity than other populations outside of Africa, and Native Americans had the lowest diversity. These trends were consistent for both nonreference and polymorphic reference insertions, except for nonreference SVAs. The relative difference in diversity between Africans and the most diverse non-African group (South Asians) was greater for polymorphic MEIs than for SNPs (supplementary table 4, Supplementary Material online). Whether this pattern reflects a technical bias or a demographic/biological process remains an open question. The rate of retrotransposition is influenced by *SAMHD1*, *MOV10*, *ZC3HAV1*, and other genes (Arjan-Odedra et al. 2012; Moldovan and Moran 2015; Goodier 2016; White et al. 2016). This suggests plausible mechanisms by which variation at these loci could produce differences in retrotransposition rates among early modern human groups with low effective population sizes. The effects of variation at these genes, individually or in combination, on MEI activity within and between current human populations remains unexplored.

We quantified the fraction of MEI diversity attributable to population subdivision (*F*_ST_) among the seven major world populations. *F*_ST_ estimates among all seven major population groups were 7.3%, 7.4%, 5.1%, and 8.1% for *Alus*, LINE-1s, SVAs, and SNPs, respectively. In comparison, the global SNP-based *F*_ST_ estimate for the 1000 Genomes Project was 8.3% (1000 Genomes Project Consortium, et al. 2010). *F*_ST_ among the four major Eurasian groups was lower (4.0%). In contrast, the highest pairwise *F*_ST_ estimates were between Africans and Native Americans (12.4% and 13.5% for *Alus* and LINE-1s) and Oceanians and Native Americans (12.4% and 13.0% for *Alus* and LINE-1s), consistent with the effects of geographic separation and genetic drift on population differentiation (supplementary tables 5*a* and *b*, Supplementary Material online). The *F*_ST_ estimates obtained here with three MEI marker systems are somewhat lower than earlier studies of MEIs (Watkins et al. 2003; Witherspoon et al. 2006), and SNPs (1000 Genomes Project Consortium, et al. 2010). A broad and uniform sampling of human populations typically provides a more accurate and lower estimate of the overall average diversity among populations (Xing et al. 2010). These results emphasize the relatively low differentiation among human groups, as expected for a rapidly expanding species with moderate gene flow among groups. In comparison, common chimpanzee populations that have little between-population gene flow can show high genetic differentiation (microsatellite *R*_ST_ = 0.31, Eastern vs. Western chimpanzee) (Becquet et al. 2007).

### Ancestry and Admixture

**Fig. 4.**
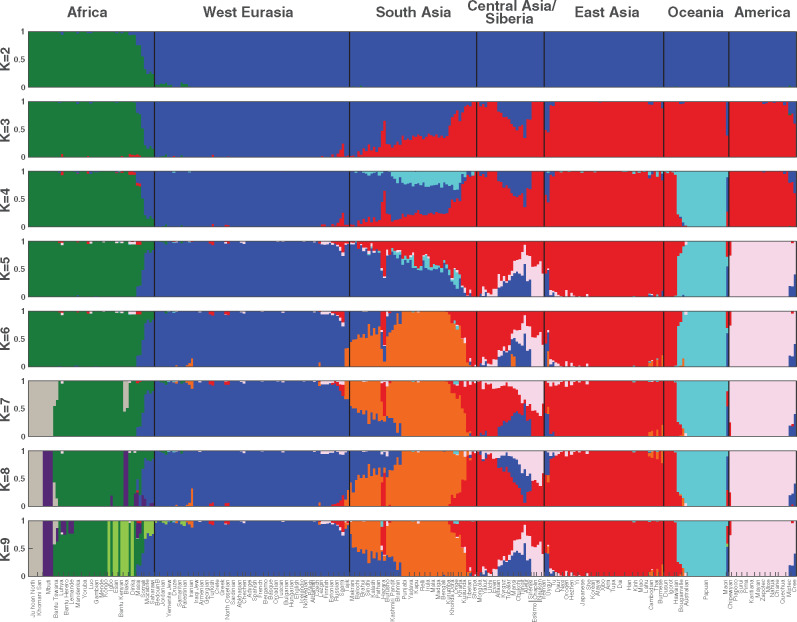
—MEI-based estimates of admixture among the SGDP populations. Admixture among the 142 populations was modeled using two to nine predicted ancestral populations and all polymorphic autosomal MEIs. K represents the number ancestral groups in the given model. Each individual is plotted as a stacked colored bar on the *x* axis, and each color in the bar shows the proportion of admixture for each of the K ancestral groups in that model. Individuals are also grouped by major geographic region. Populations bordering transitions between Africa and West Eurasia, South Asia and West Eurasia, and East Asia and South Asia are substantially admixed. Many Central Asian and Siberian populations demonstrate tri-partite shared ancestry consisting of Asian, European, and New World components.

We modeled ancestry and admixture in the SGDP using the combined set of nonreference polymorphic *Alu*, LINE-1, and SVA MEIs (fig. 4). Models with a low number of predicted ancestral groups (K2–K5) show that ancestry estimates are largely apportioned by major geographic region (i.e., Africa, West Eurasia, East Asia, Oceania, and the Americas). One exception to this pattern was the Papua New Guineans, who form a distinct group and have notable affinity with the Onge and populations from South India at K4 (cyan). As the predicted number of ancestral groups increases (K6–K9), South Asian populations become distinct from other world populations (orange) and are characterized by West Eurasian admixture (blue) in some populations living in northwest India, Kashmir, Pakistan and neighboring locales. This finding is consistent with geographically structured gene flow into the northern regions of India but limited gene flow into southern India (Reich, Thangaraj, et al. 2009; Narasimhan et al. 2019).

Within Africa, the isolated San/Ju’hoan and Mbuti pygmy populations of southern Africa each form a distinct ancestral group (K8 and K9), and there is evidence of gene flow between the Mbuti (purple) and neighboring groups (Luhya, Herero, and Tswana). There is shared ancestry among some equatorial African populations including the Esan (Nigeria), Bantu-speaking Kenyans, Dinka (Sudan), Biaka (CAR), Somali, and Maasai (Kenya) (*K* = 9, light green component). This ancestry component is also present in some northern Africans. Saharan populations, such as the Saharawi (Morocco) and Mozabite (Algeria), however, are characterized by western Eurasian ancestry. These results, combined with lower heterozygosity and a smaller number of private alleles in the Saharawi and Mozabites, emphasize the critical role of geographic barriers such as the Saharan desert in shaping the MEI diversity patterns in modern humans (Fan et al. 2019) (see also fig. 3).

### New World Shared Ancestry

**Fig. 5.**
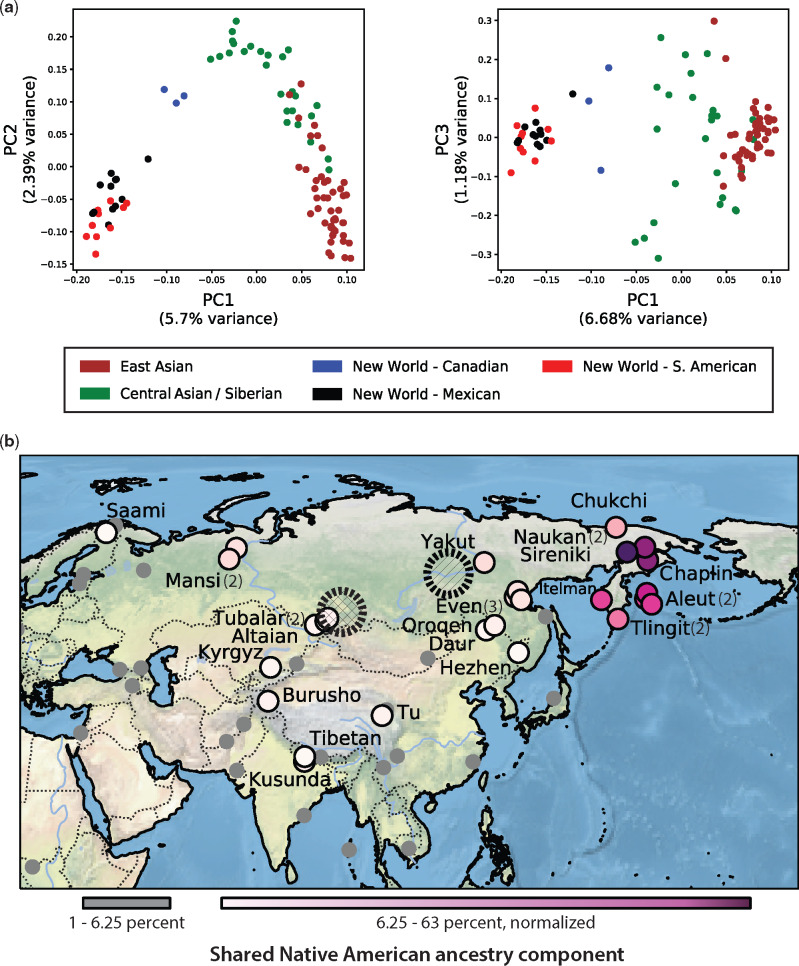
—MEI estimated Native American ancestry in Asia. (*a*) PCA of the genetic relationship among East Asians (46), Central Asians/Siberians (26), and Native Americans (26) based on all nonreference *Alu*, LINE-1, and SVA elements. Panel 1: PC1 separates New World samples from Asian samples. Native Americans are separated along PC2 and trend toward Central Asians (Siberians) according to their geographic location in the Americas. Panel 2: PC3 separates Central Asians, Siberians, and Canadian Native Americans. The primary genetic affinity of New World samples remains closer to Central Asians than to East Asians along PC1. (*b*) The geographic distribution of all Asian individuals with >1% Native American shared ancestry. Colored dots (pink–purple) show individuals with 6.25–63% New World shared ancestry. Population names and the number of individuals, if more than one, are indicated. Small uniform gray dots show other individuals with low, but detectable, New World shared ancestry (1–6.25%). The geographic centroid for all Old World individuals with Native American shared ancestry is indicated by the cross-hatched circle. The geographic centroid based only on individuals with >6.25% New World ancestry is indicated by the hatched circle.

Ancestry for New World individuals in the SGDP is partitioned predominantly into a stable unique ancestry component (see fig. 4, K5–K9, pink). This New World ancestry component is not seen in most eastern Asians but is found at appreciable frequency in individuals from Siberia and central Asia. PCA of Native Americans, Central Asians, Siberians, and eastern Asians shows that, on a genome-wide scale, the primary genetic affinity of New World populations is to Central Asians rather than East Asians (fig. 5*a*).

To better resolve the Central Asian genetic signal present in populations from the Americas, we examined the fraction of shared ancestry and geographic location of all Old World individuals with detectable (>1%) New World shared ancestry. As expected, given their proximity to North America, we find high levels (54–63%) of the New World shared ancestry component in the Chaplin, Sireniki, and Nauken populations of extreme northeastern Asia. Of greater interest is the finding that populations sampled in mainland Siberia such as the Yakut, Even, Tubalar, and Mansi maintain up to 15% Native American shared ancestry. Other populations of central Asia also have 5–15% of this ancestry component, but, as the distance from Beringia to the south and west increases, New World shared ancestry decreases.

We used the geographic locations of all Old World individuals with >1% New World shared ancestry to calculate a geographic centroid within Asia for the Native American shared ancestry component. The centroid (cross-hatched circle) occurs in Khakasiya, Siberia (53.92 latitude, 90.66 longitude) and is bordered on the South by the Altai and Tyva regions (fig. 5*b*). Using only individuals with substantial New World ancestry (>6.25%) moves this centroid to the northwest into the upper Baikal and Lena river regions (60.47 latitude, 119.08 longitude). Due to its remoteness, central Siberia remains undersampled, and additional populations could further refined these estimates. Our results, based genome-wide strictly identical-by-descent MEI insertions, provide a robust inference of a southern to southeastern Siberian origin for the primary wave of early migrants that gave rise to most Native Americans today. This shared ancestry signal likely corresponds to the “First Americans” migration wave, which is one of at least three migration waves contributing to New World populations (Reich et al. 2012).

Mitochondrial and Y-chromosome analyses also support a common ancestral population for Native Americans and southern Altaians (Dulik et al. 2012). Recently, several Y-chromosome Q1 lineages from southern Siberia have been linked to founding Native American lineages and have provided an estimated migration date of at least 14.3 kya (Wei et al. 2018). Our results substantially strengthen these conclusions because 1) they are based on strictly identical-by-decent markers, 2) the estimates are made using a broad sampling of Asian populations, and 3) using thousands of markers reduces the effects of sampling variation and genetic drift that may influence estimates based on single markers. Though lacking the power and resolution of a full genome-wide SNP analyses, our results are in accord with an analysis of autosomal markers from two ancient central Asian DNA samples which suggest that an ancient southern Siberian population split from ancestral eastern Asians and then gave rise to New World populations (Raghavan et al. 2014). Our results are also consistent with recent genome-wide data from 48 ancient samples that map Paleo-Eskimo shared ancestry in North American Na-Dene and Aleuts speakers to a Siberian origin (Flegontov et al. 2019).

Our estimate of the geographic centroid for Native American shared ancestry within Asia is based on a small sample of modern Native Americans (26 individuals from 13 populations). Therefore, the inference and resolution of the Native American ancestry components is also limited by sampling. For example, the finding of populations in this study with low levels of Native American shared ancestry such as the Onge and other southeast Asian groups is consistent with additional ancestral groups of Asian founders that have contributed shared ancestry to some Native South Americans, as previously reported (Skoglund et al. 2015). Removing samples with very low Native American ancestry substantially shifts our estimate of the shared ancestry centroid northwest into the Baikal/Lena region, in better agreement with some studies. Several studies have found evidence for two to four detectable migration or gene flow events in Native Americans (Reich, Thangaraj, et al. 2009; Skoglund et al. 2015). Although evidence of population substructure in ancient Siberian samples has been reported (Yang et al. 2017), we did not attempt to discover multiple shared-ancestry locations within Asia. Recent work examining numerous ancient samples from the Americas now appears most consistent with early lineages diverging within the Americas followed by complex migration and admixture events (Raghavan et al. 2015; Moreno-Mayar et al. 2018; Posth et al. 2018; Flegontov et al. 2019).

### MEI Subfamily Analysis

Only a small subset of MEI insertions can give rise to new retrotransposition events. Active LINE-1 elements are necessary for most MEI retrotransposition, but active LINE-1 elements may vary in frequency among individuals and populations. Previous studies have reported substantial differences in the retrotransposition activity of full-length LINE-1 elements (Brouha et al. 2003; Beck et al. 2010). To determine if asymmetry in the distribution of active LINE-1 insertions among populations has produced variation in the distribution of MEI subfamilies, we examined the distribution of polymorphic nonreference *Alu* and LINE-1 subfamilies in aggregate and among major populations groups.

**Fig. 6.**
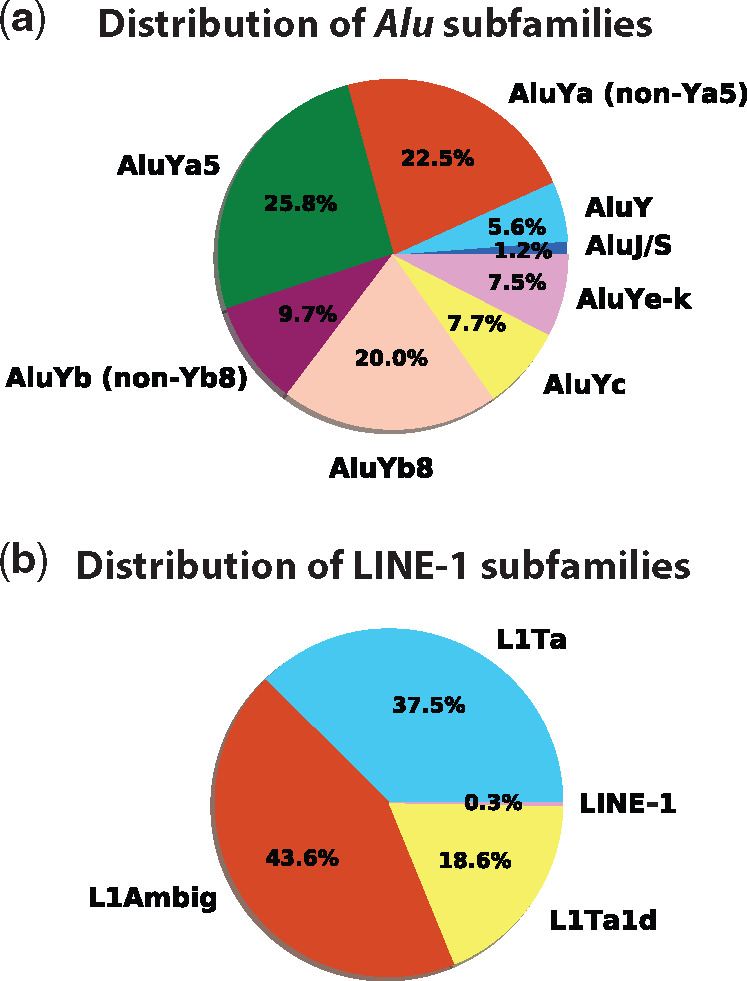
—Distribution of nonreference *Alu* and LINE-1 families found in the SGDP populations. Pie charts show the percentages of the major families identified in the SGDP. (*a*) The frequencies of aggregated *Alu* families and (*b*) the frequencies of aggregated LINE-1 families (see supplementary tables 6*a*–*c* and 7, Supplementary Material online, for nonaggregated results).

For MEI data aggregated by major MEI subtype (see Materials and Methods) over all populations, *Alu*Ya5, *Alu*Ya, and *Alu*Yb8 insertions accounted for 25.8%, 22.5%, and 20.0% of new *Alu* insertions, respectively. L1 (subfamily indeterminate) and L1Ta insertions made up the majority (43.6% and 37.5%) of new LINE element insertions (fig. 6**)**. The insertion frequencies of the aggregated subfamilies were very similar across populations. In general, the frequencies of the aggregated subfamilies varied <±6% among the seven major population groups (supplementary table 6*a*–*c*, Supplementary Material online). We also examined the unaggregated subfamily data for all *Alu* loci and found that 51 of 63 subfamilies (81%) were present in all seven major population groups (supplementary table 7, Supplementary Material online). The overall distribution of these subfamily frequencies was not significantly different among populations (*P* ≥ 0.99, one-way ANOVA). For the 12 A*lu* subfamilies not shared among all seven major populations, Africans had the most subfamilies (10/12), whereas Native Americans had the fewest population-specific subfamilies (2/12). Only three subfamilies were limited to single populations (Africa: *Alu*Yb3a2, Western Eurasians: *Alu*Yh9, and Central Asians/Siberians: *Alu*Yi6). Together, these findings indicate that most polymorphic *Alu* subfamilies (∼80%) predate the expansion of modern humans from Africa.

### Subfamily Haplotype Diversity

To characterize the diversity and evolutionary dynamics of *Alu* subfamilies, sequences at each *Alu* locus in every individual were assembled, de novo, from short-read sequencing data. Identical consensus sequences were successfully generated for 5,687 loci using two independent approaches (see Materials and Methods). These de novo assemblies were then used to examine *Alu* subfamily diversity. We used loci that were found in at least ten independent chromosomal locations to ensure that each *Alu* element represents a sequence for an active *Alu* subfamily. Consensus sequences were assigned to known subfamilies based on established classification criteria (Batzer and Deininger 2002; Konkel et al. 2015; Feusier et al. 2017; Gardner et al. 2017).

**Fig. 7.**
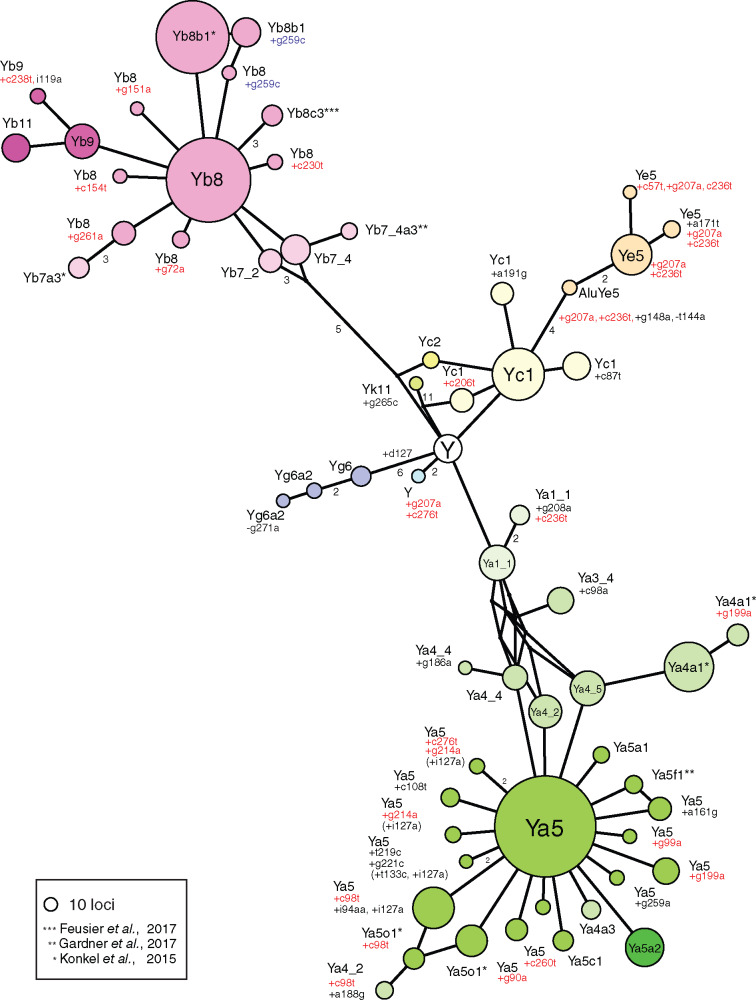
—Median-joining network of active *Alu* subfamilies. A median-joining network of full-length *Alu* elements discovered from 296 fully sequenced genomes illustrates their relationships and relative proportions in this diverse set of world populations. The star-like topologies for the *Alu*Ya5 and *Alu*Yb8 indicate recent rapid expansion of these subfamilies. The highly variable middle A-rich region and poly(A) tail were masked for each sequence prior to clustering. Each node represents an *Alu* haplotype that was found in at least ten independent loci. The number of segregating loci identified is proportional to a node’s radius. Additional mutational changes from each known subfamily are specified at the nodes, and most are transitions at CpG sites (red). Some *Alu*Yb8 nodes have mutations within a diagnostic indel. These mutation locations are based on the *Alu*Yb8 consensus (blue) rather than the *Alu*Y consensus (black). A node may also contain haplotypes with additional mutations within the masked middle A-rich region for the haplotype assignment, and these are indicated parenthetically. Edges represent one classifying mutational step unless otherwise specified. The Y node located in the center is the *Alu*Y consensus sequence.

A radial network of these active subfamilies shows three major *Alu* clades (*Alu*Ya, *Alu*Yb, and *Alu*Yc) emanating from a central *Alu*Y-consensus node (fig. 7). Significant reticulations of the *Alu*Ya4 subfamilies lead to the very common *Alu*Ya5 subfamily, which has generated 17 active descendant subfamilies that differ from the parent by one to several key mutations. The *Alu*Yb8 and *Alu*Yc1 also branch independently from the Y-consensus and have similar patterns of diversity but fewer descendent nodes. The *Alu*Ya5 and *Alu*Yb8 subfamilies demonstrate star-like topologies. These topologies, the occurrence of *Alu*Ya5 and *Alu*Yb8 families and derivative subfamilies in all major population groups, and negative estimates of the Tajima’s *D* statistic (table 3) are consistent with the effects of purifying selection and a continuing expansion of the *Alu*Ya5 and *Alu*Yb8 subfamilies in all human populations. Several subfamilies, such as the *Alu*Yb9, *Alu*Ye5, and *Alu*Ya4a1, also show independent but limited activity and have given rise to a small number of active subfamilies.

**Table 3:** Sequence-based Diversity Estimates for Major *Alu* Subfamilies

	Sequences	Haplotypes	Segregating Sites	π	Θ	Tajima’s *D*
*Alu*Ya5	541	67	24	0.010597	0.016868	−0.24204
*Alu*Yb8	892	68	24	0.006404	0.016376	−0.96653
*Alu*Yc1	256	25	6	0.005751	0.005405	1.42585

Note.—π, observed average pairwise diversity among sequences; Θ, expected diversity among sequences.

## Materials and Methods

### Study Samples

The SGDP data set consists of 300 world samples from seven major population groups, 75 geopolitical regions, 142 populations, and has been described previously in detail (Mallick et al. 2016). Informed consent was obtained for all individuals. Previously generated WGS data were downloaded from the European Nucleotide Archive (PRJEB9586/ERP010710) or obtained directly from the authors of the original study. Sequencing reads (HiSeq, 100-bp paired-end reads) from each sample were aligned to the human reference sequence (build 38) using the BWA-MEM algorithm (ver. 0.7.12). The average depth of coverage for all realigned samples was 43.12 (range 31.97–83.23). SNP data for each sample were obtained in variant call format files (Mallick et al. 2016). SNP data were filtered to include only sites with quality scores from 1 to 9, and build 37 positions were lifted to build 38 with the UCSC liftOver tool. To allow direct comparisons of MEI and SNP data sets, four samples were removed due to 1) insufficient paired reads (<95%) for MEI genotyping or 2) missing SNP filter quality scores, yielding a final data set of 296 samples (see supplementary table 1, Supplementary Material online).

### MEI Identification and Genotyping

To obtain the locations and genotypes of nonreference *Alu*, LINE-1, and SVA MEIs, samples were analyzed with the MELT software package (ver. 2.1.4) (Gardner et al. 2017). Whole genomes of 296 SGDP samples were processed using MELT protocols on two 64-core servers with standard calling parameters and the supplied MEI-priors files. Specifically, for each MEI type, samples were jointly genotyped together along with one three-generation Centre d’Etud du Polymorphisme Humain family (http://www.cephb.fr/en/familles_CEPH.php; last accessed May 04, 2020). Nonreference MEIs were called with MELT-SPLIT program and reference MEIs were genotyped using MELT-Deletion program using the recommended standard calling procedures (see https://melt.igs.umaryland.edu/manual.php; last accessed May 04, 2020). Known MEIs from the hg38 reference were identified using the bed file provided in the MELT package. Additional filters were employed to reduce false positive calls and genotyping errors. Specifically, only MELT PASS loci with MELT-indentified target site duplications on either side of the MEI (MELT ASSESS = 5) were retained. MEI loci that failed HWE in any of the seven major population groups also were removed (*P* ≤ 0.05, Bonferroni corrected). For each type of MEI insertion, the majority (∼98%) of loci excluded by the HWE screen were due to a large excess of heterozygotes, suggesting genotyping issues at those loci. The HWE screening step excluded ∼1–4% of potential MEIs, depending on the MEI type; 83 MEIs were removed. HWE filtering improved the overall genotype quality in the final data set while modestly increasing the false negative rate. Therefore, our discovery rate estimates are likely to be conservative. Additional details of the MEI discovery and calling process are presented in the Supplementary Material (see supplementary table 2, Supplementary Material online). The MELT-Deletion algorithm was used to call and genotype MEI presence–absence polymorphisms in the reference sequence. MELT-transduction was used to identify LINE-1 transduction events.

To compare insertions found in the SGDP to the 1000 genomes, MEI data were downloaded from the 1000 Genomes Project (http://ftp.1000genomes.ebi.ac.uk/vol1/ftp/phase3/integrated_sv_map/ALL.wgs.mergedSV.v8.20130502.svs.genotypes.vcf.gz; last accessed May 04, 2020). Locations from the data sets were intersected after lifting positions to hg38. Although many MEI insertions were found to have identical locations between these two call sets, the number of overlapping MEIs was substantially increased by allowing a match to a windowed region of ±25 bp (see supplementary fig. 3, Supplementary Material online). The minor variation in location was due primarily to different versions of MELT used in the two analyses.

### MEI Locations, Heterozygosity, and Transcriptional Orientation

To obtain locations and regions of interest for analysis of MEIs with respect to the build 38 genome sequence, we used the conservative RefSeq (build 38) definitions of known exons and genes. For protein-coding regions, potential MEI insertion locations from the MELT output were intersected with RefSeq coding regions. All potential protein-disrupting MEIs were then verified manually using the Integrated Genome Viewer.

Heterozygosity and the number of private alleles for each individual were calculated using vcftools (v0.1.13). Individual heterozygosity and private allele values were plotted on Robinson map projections using the matplotlib basemap or cartopy packages implemented in the Julia programing language. Heterozygosity for major groups was estimated as the average heterozygosity over all loci.

The transcriptional orientation of each gene was downloaded from the UCSC genome database. A gene’s transcriptional orientation was then compared with the insertional orientation of the MEI as designated by the MELT analysis and classified as being in the same or opposite as that of the gene. Significance was evaluated by a generalized *z*-test of proportions.

### Genetic Structure Analyses

Genetic distances among individuals were calculated as allele-sharing distances, and PCA was performed using the SNPRelate package for R (ver. 1.6.6) (Zheng et al. 2012). *Alu*, LINE-1, SVA, and SNP data for the PCA included only common autosomal loci (minor allele frequency ≥0.02) that were not in linkage disequilibrium (*r*^2^ ≤ 0.2) and were typed in at least 90% of all samples. For SGDP SNP data (Mallick et al. 2016), polymorphisms at all CpG sites were removed to eliminate possible identity by state sites. Genetic distances among major population groups were also calculated using Nei’s standard distance, and neighbor-joining networks were created using the PHYLIP package (Felsenstein 2005). Admixture among individuals was estimated using the ADMIXTURE software package (Alexander et al. 2009). *F*_ST_ values were calculated using Weir and Cockerham’s method as implemented in the vcftools software package. Individual MEI heterozygosity estimates were calculated as the fraction of observed heterozygous sites in the nonreference MEI discovery data sets for each individual.

Geographic distances among populations were calculated as great circle distances using the haversine formula. For worldwide geographic distance analyses, distances to the African centroid assumed that human migrations from Eurasia into the Americas occurred via a Beringian route. Thus, distances for the New World samples were calculated as the sum of two great circle distance segments, from the African centroid to Naukan, Russia (66.027222, 169.7077782), the former eastern-most settlement point of Eurasia, and then to the New World population.

For the analysis of Native American ancestry, the fraction of Native American ancestry was estimated across all samples using *K* = 7 predicted groups. Old World populations were divided into those with substantial Native American ancestry (6.25%) and those with lower ancestry (1–6.25%). For perspective, the estimated Neanderthal admixture in non-Africans is typically between 1.5% and 2.1% (Prüfer et al. 2014). We then calculated the geographic centroid of: 1) all Old World individuals or 2) Old World individuals with >6.25% Native American admixture within Asia as described above.

### MEI Family and Subfamily Analysis

The assignment of newly identified MEI insertions to subfamilies was performed using the MELT program. To reduce the number of rare *Alu* subfamilies with low counts for some analyses, low-frequency subfamilies were combined. *Alu*Ye-k were aggregated, as were *Alu*Yb and *Alu*Ya inserts that were non-Yb8 or 6 and nonYa5, respectively. *Alu* families and subfamilies were analyzed directly or aggregated into major family types. LINE-1 subfamilies were analyzed directly without aggregation. SVAs were not subtyped by MELT. The MEI families were analyzed over the whole data set and for each major population grouping. Additionally, we attempted de novo assembly for all nonreference *Alu* insertions in all individuals using an in-house method and a modified subroutine of the TE-type algorithm (Goubert et al. 2020) specifically designed for generating de novo assemblies for nonreference *Alus* (https://github.com/jainy/Non-reference-Alu_Assembly; last accessed May 04, 2020). A total of 5,687 consensus sequences were identical using both approaches, and these loci were retained for more detailed subfamily analyses. The assembled *Alu* sequences were also compared with their MELT subfamily assignments and were generally concordant (79%), but some loci and subfamilies (e.g., *Alu*Yb6_2, *Alu*Ye) were more accurately assigned using the assembled consensus sequences. Many nonconcordant assignments were attributable to variation within an indel near the 3′ end of the element. *Alu* subfamilies were compared with one another using a median-joining network as implemented in the POPART software package (Leigh and Bryant 2015). Nucleotide diversity within subfamilies was estimated as the average pairwise difference among all sequences (π) and was compared with a neutral expectation of diversity (Θ) using Tajima’s *D* statistic.

## Supplementary Material

evaa086_Supplementary_Data

## Notes

**Data deposition:** Sequencing data for 279 samples can be obtained from the EBI European Nucleotide Archive (accession numbers: PRJEB9586 and ERP010710). Data for an additional 21 genomes are available by signed-letter access from the European Genome-Phenome Archive (EGA) Data Access Committee. All variant call format files of the mobile element insertion (MEI) calls and analysis code are available from the authors on request or may be downloaded from Supplementary Material online.
