# Supplementary material for: The Simons Genome Diversity Project: A Global Analysis of Mobile Element Diversity
Source: Genome Biol Evol. 2020 May 2;12(6):779–94. doi: 10.1093/gbe/evaa086 (PMC7290288; doi:10.1093/gbe/evaa086)
Supplement: evaa086_Supplementary_Data [file evaa086_supplementary_data.zip › SGDP_Watkins_etal_GBE200212_SupplementalMaterial_minor_revisions.docx]

**Supplementary Material**

*Study individuals*

The goal of SGDP sampling strategy was to maximize genetic diversity. For analytical purposes, samples were divided into seven major geographic regions. Each region contains a minimum of 25 people. Four samples were removed from the original 300 Simons Genome Diversity Project (SGDP) due to sample processing issues and matching MEI and SNP data sets. A description of all samples used in this study is shown in **Supplementary Table 1**.

| **Supplementary Table 1. Study samples listed by geopolitical region** | | | |
| --- | --- | --- | --- |
| **Region** |  | **N** |  |
| **Africa** |  | **49** |  |
| Algeria 2, Botswana/Namibia 4, Cameroon 2, Central African Republic 2, Congo 5, Gambia 2, Kenya 9, Namibia 4, Nigeria 7, Senegal 3, Sierra Leone 2, South Africa 2, Sudan 3, Morocco 2 | |  |  |
| **America** |  | **26** |  |
| Argentina 1, Brazil 5, Canada 3, Colombia 2, Mexico 12, Peru 3 | |  |  |
| **Central Asia and Siberia** |  | **26** |  |
| China (Mongolia) 2, Kyrgyzstan 2, Russia 22 | |  |  |
| **East Asia** |  | **46** |  |
| Cambodia 2, China 30, Japan 3, Korea 2, Myanmar 2, Taiwan 3, Thailand 2, Vietnam 2 | |  |  |
| **Oceania** |  | **25** |  |
| Australia 2, Brunei 2, New Zealand 1, Papua New Guinea 17, Philippines 2, USA (Hawaii) 1 | |  |  |
| **South Asia** |  | **49** |  |
| Bangladesh 2, India 21, Nepal 4, Pakistan 20, Tibet 2 | |  |  |
| **West Eurasia** |  | **75** |  |
| Abkhazia 1, Albania 1, Armenia 2, Bulgaria 2, Czechoslovakia 1, England 2, Estonia 2, Finland 5, France 5, Georgia 2, Greece 4, Hungary 2, Iceland 2, Iran 2, Iraq 2, Israel 1, Israel (Carmel) 2, Israel (Central) 3, Israel (Negev) 2, Italy 1, Italy (Bergamo) 2, Italy (Sardinia) 2, Italy (Tuscany) 2, Jordan 3, Norway 1, Orkney Islands 2, Poland 1, Russia 8, Russia (Caucasus) 2, Spain 2, Tajikistan 2, Turkey 2, Yemen 2 | |  |  |
| **Total** |  | **296** |  |

*MEI discovery in the SGDP samples*

MEIs were discovered using the MELT software package (version 2.1.4). To obtain a high-quality data set with the best possible genotypes, the raw MELT calls were stringently filtered. We used three major inclusion criteria to refine the quality of MEIs included in the final data sets. First, all potential MEI loci were required to have a PASS tag by MELT. Second, all MEIs were required to have target-site duplications (TSD) as defined by MELT on each side of the insertion. Flanking TSDs are a hallmark of the retrotransposition process, and the TSD requirement ensures that MEIs included in the final data sets are consistent with known retrotransposition events at the molecular level. MEIs were required to have a top MELT ASSESS score of five. Finally, all MEIs were required to be in Hardy-Weinberg equilibrium in each of the seven major populations (Africans, Americans, Central Asians/Siberians, East Asians, Oceanians, South Asians, and West Eurasians) represented in the Simons Genome Diversity Project (SGDP) collection. The HWE filtering step removes 1.4 - 4.2% of potential MEI insertions (288 MEIs). The strict inclusion criteria increase the false negative rate but reduce the number of MEI loci that are poorly genotyped, thus improving the overall quality of the final *Alu*, LINE-1, and SVA data sets for downstream applications.

| **Supplementary Table 2. Quality filtering for MEI discovery data sets** | | | |
| --- | --- | --- | --- |
|  | ***Alu*** | **LINE-1** | **SVA** |
| Quality Filters |  |  |  |
| Raw calls (no filters) | 102,444 | 25,117 | 5884 |
| PASS loci | 13,052 | 2327 | 909 |
| MELT ASSESS=5 | 11,968 | 1925 | 500 |
| HWE  (p < 0.05, Bonferroni corrected) | 11,727  (-2.1%) | 1899  (-1.4%) | 479  (-4.2%) |
| AC0 filter | 11,661 | 1886 | 475 |
| **Final count** | **11,661** | **1886** | **475** |

(Numbers show the loci remaining after each filtering step.)

***Identification of SGDP MEIs in the 1000 genomes (phase 3) data***

MEIs discovered in the SGDP samples were compared to MEIs discovered in the 1000 genomes phase 3 (1K genomes) samples. The 1K genomes collection has 2504 samples from 26 diverse world populations. Many MEIs discovered in the two data sets had identical positions (1K genomes lifted to Hg38). Allowing a small window of positional tolerance (± 25 bp) increased the overlap increased substantially (**Supplementary Figure 1**). Examination of the positional differences between the two data sets revealed that the differences could be attributed to TSD length and the corresponding MEI start and stop positions assigned by the different versions of MELT.

**Supplementary Figure 1.** **Number of MEIs discovered in the SGDP that intersect with the 1K genome phase 3 data set as a function of window size.** A plateau in the number of MEIs found in both data sets occurs at ± 20 - 25 bp. A window value of ± 25 bp was used for the final MEI comparisons with the 1000 genomes.

*Insertion size and target site duplication length*

**a.**

**b.**


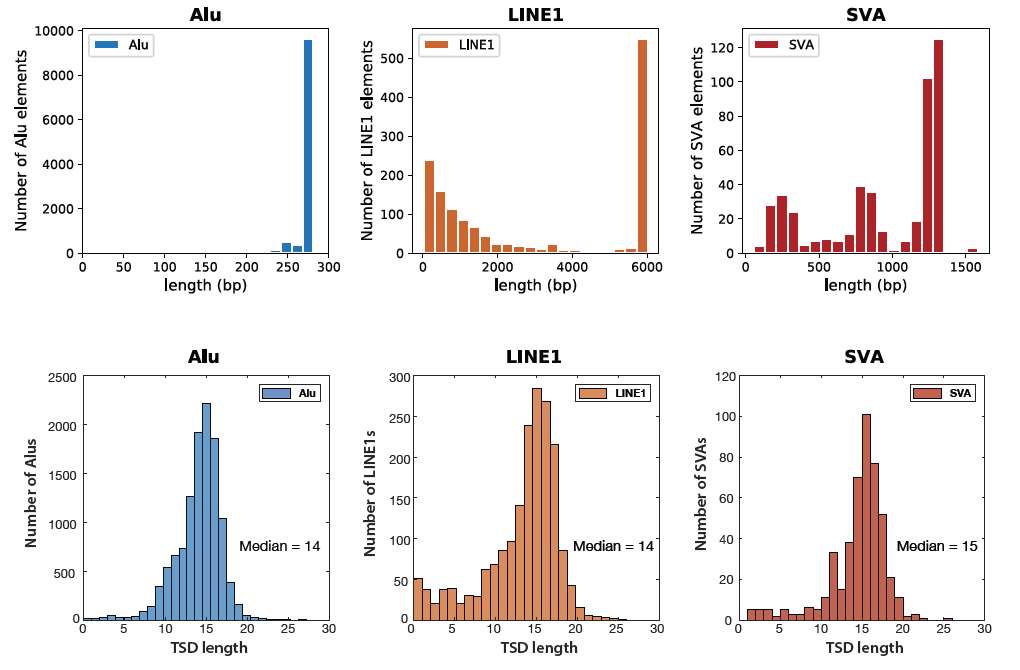


**Supplementary Figure 2. Insertion size and TSD length for *Alu*, LINE-1, and SVA elements**

**a)** The distribution of MEI lengths for *Alu*, LINE-1, and SVA elements. Most *Alu* elements are full-length. Nearly half (46%) of LINE-1 elements are characteristically truncated. Most SVA elements are also truncated. **b)** The distribution of target site duplications lengths found at MEI insertion sites. *Alu*, LINE-1, and SVA elements have similar TSD length distributions with a median TSD of ~14 – 15 bp. Similarity in TSD length among MEI classes is likely attributable the common mechanism by which new elements are generated, LINE-1-ORF2p mediated retrotransposition.

*MEI events disrupt genes*

MEIs occassionally insert into coding regions of genes. These events disrupt target genes because the MEI introduces sequence that typically creates a new termination codon soon after the insertion site. For most genes, the early termination is detrimental, and these new exonic MEI insertions are eliminated from the population by negative selection. To characterize the effects of MEI retrotranspostion on the coding regions of SGDP individuals, all MEI elements identified in the SGDP were intersected with the currated RefSeq genes. A total of 10 SGDP-specific MEIs were identified in verified protein coding regions (**Supplementary Table 3**, **Supplementary Table 4**). Inline with expectations of purifying natural selection, the frequency of these insertions were very low, and most insertions are found as heterozygotes in only one or two individuals.

| **Supplementary Table 3. MEI insertions in protein coding regions** | | | | | | | | | | |
| --- | --- | --- | --- | --- | --- | --- | --- | --- | --- | --- |
| MEI | Location (hg38) | Strand  (MEI/gene) | Het,Hom  counts | | MAF | | Population group(s) | Gene | Gene information | |
| *Alu* | 2:29133663 | +/+ | | 1,0 | | 0.003 | O | CLIP4 | CAP-Gly Domain Containing Linker Protein |  |
| *Alu* | 3:150659919 | -/- | | 2,0 | | 0.007 | AF (2) | ERICH6 | Glutamate Rich 6 / testis expression |  |
| *Alu* | 4:73483970 | +/+ | | 1,0 | | 0.003 | AF | AFM | Vitamin E binding protein |  |
| *Alu* | 5:131436058 | -/- | | 1,0 | | 0.003 | AF | RAPGEF6 | Rap guanine nucleotide exchange factor 6, RAS signalling |  |
| *Alu* | 8:100573918 | -/- | | 5,0 | | 0.017 | EA, CAS (2), AM (2), | SNX31 | phosphatidylinositol binding |  |
| *Alu* | 11:7695684 | +/- | | 18,1 | | 0.068 | AF (18),  O (2) | OVCH2 | Ovochymase 2 / protease |  |
| *Alu* | 12:55426958 | +/+ | | 1,0 | | 0.003 | AF | OR6C76 | Olfactory receptor |  |
| *Alu* | 15:39829110 | +/- | | 2,1 | | 0.014 | AF (4) | GPR176 | G Protein-Coupled Receptor 176, circadian rhythm |  |
| *Alu* | 15:79898993 | +/- | | 1,0 | | 0.003 | O | ST20 | Suppressor Of Tumorigenicity 20 / cervical cancer |  |
| *Alu* | 22:46313927 | +/+ | | 1,0 | | 0.003 | AM | GTSE1 | G2 And S-Phase Expressed 1 / cell cycle |  |
| MEI – mobile element insertion; MAF – minor allele frequency; AF – Africans, AM- Native Americans, CAS – Central Asians and Siberians, EA – East Asians, O – Oceanians. | | | | | | | | | |  |


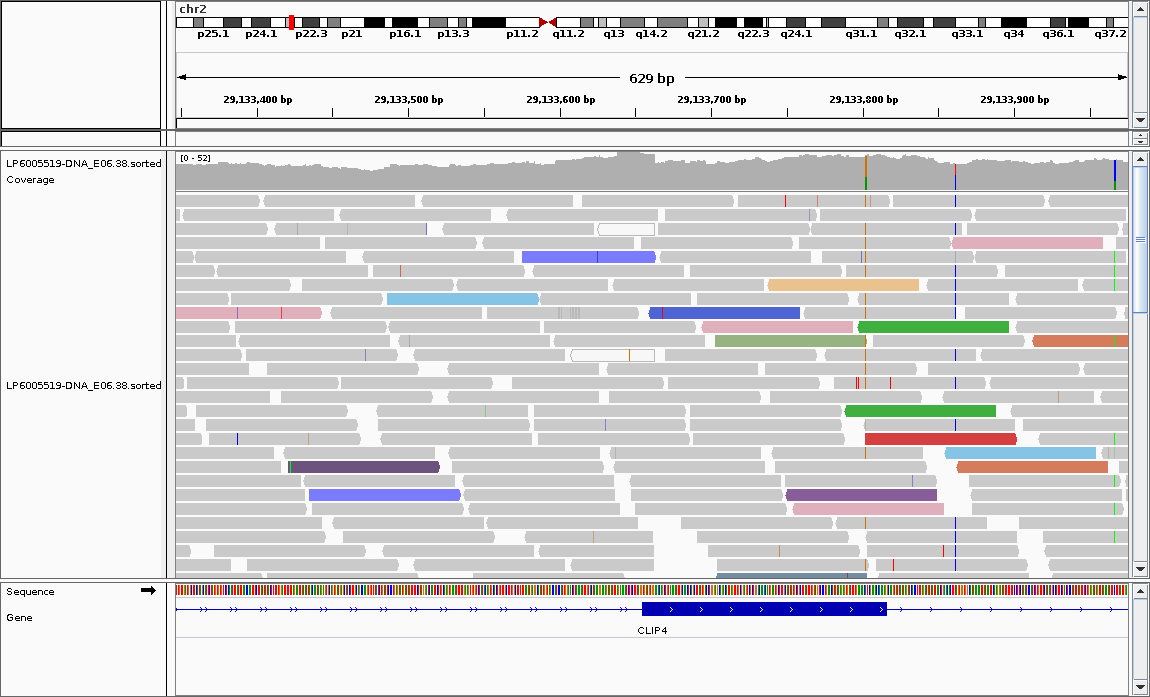


LP6005519-DNA_E06_chr2:29133663

CLIP4


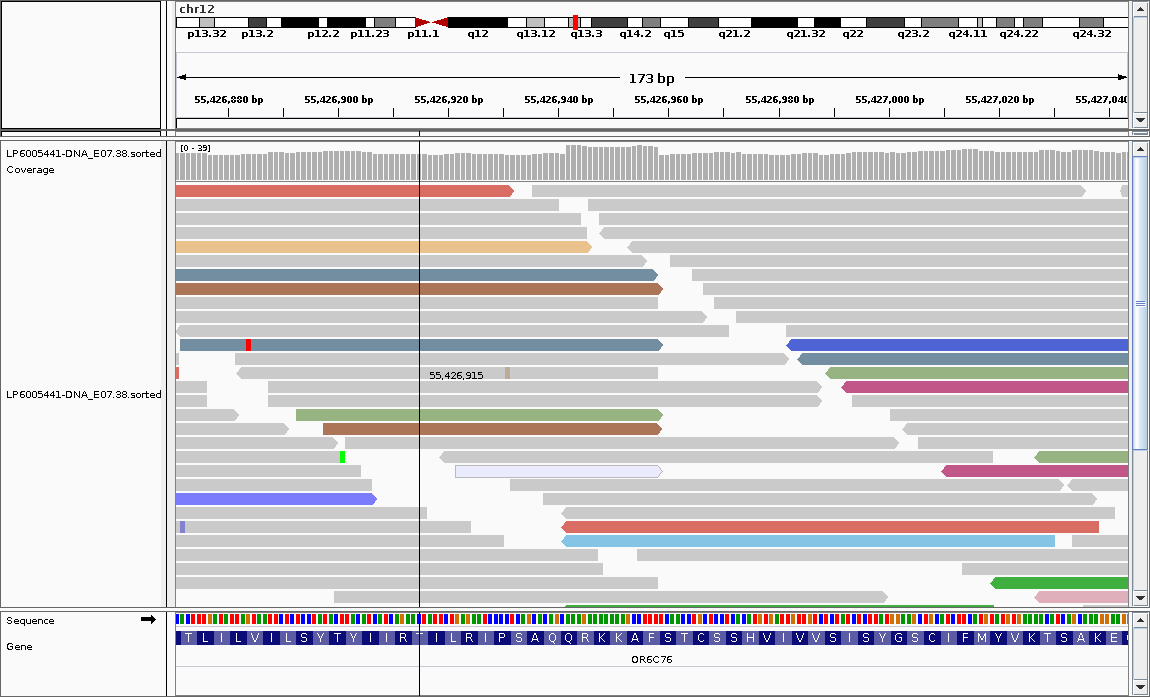


LP6005441-DNA_E07_chr12:55426958

OR6C76


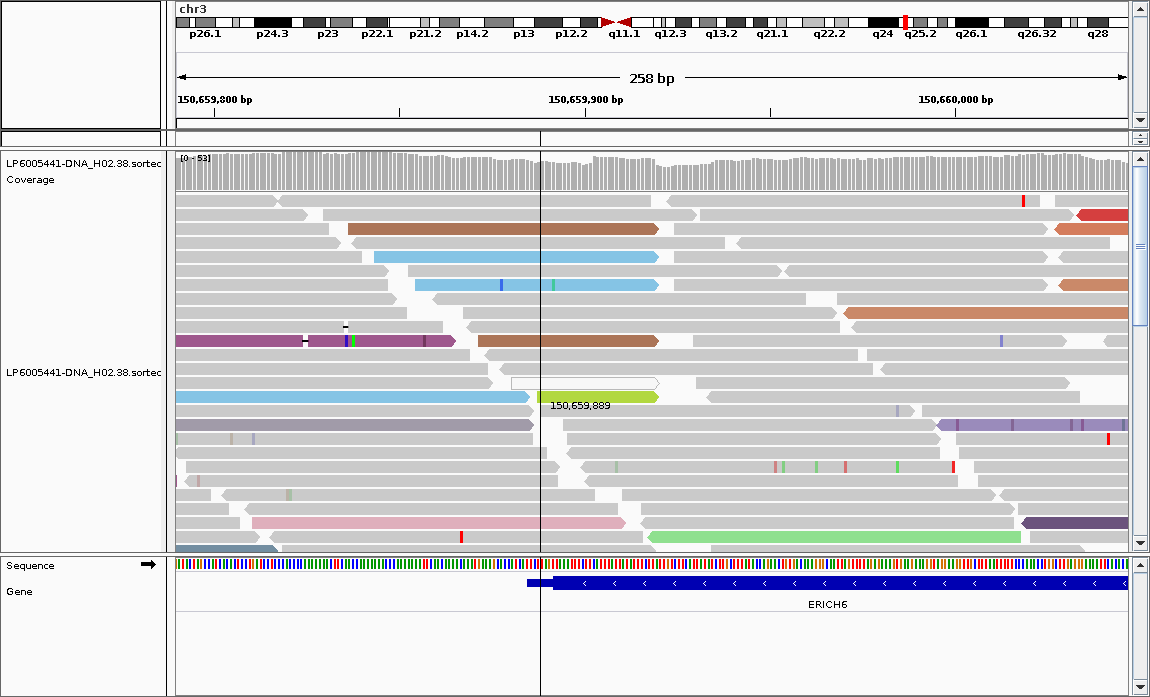


LP6005441-DNA_H02_chr3:150659919

ERICH6


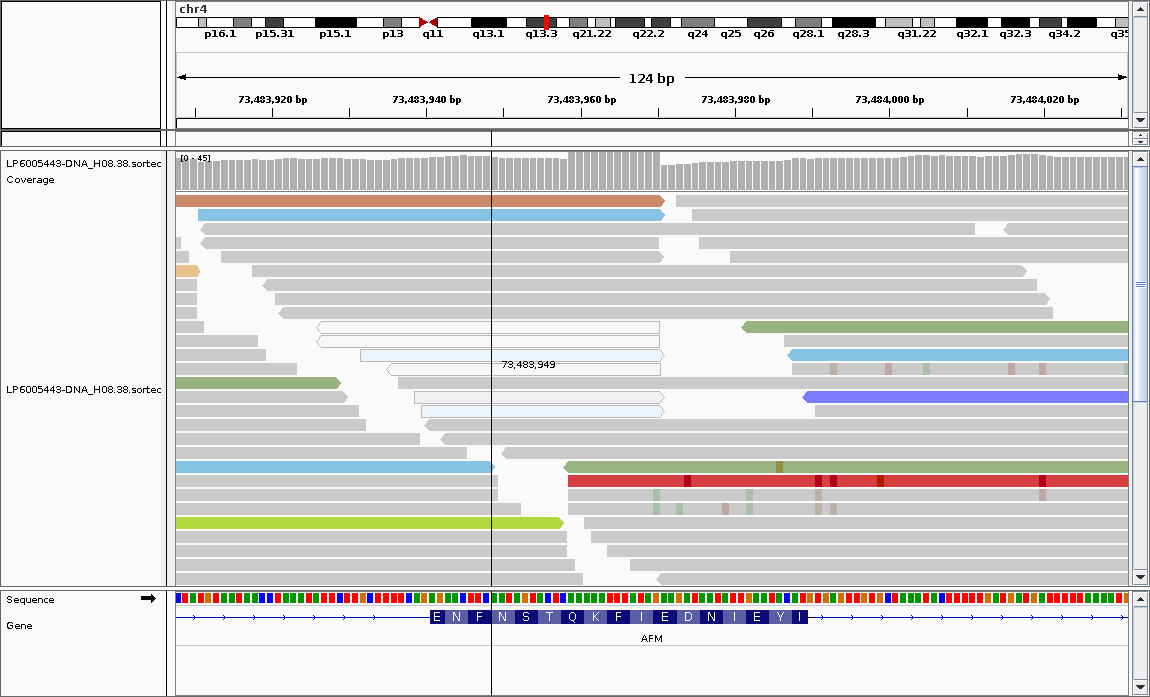


LP6005443-DNA_H08_chr4:73483970

AFM


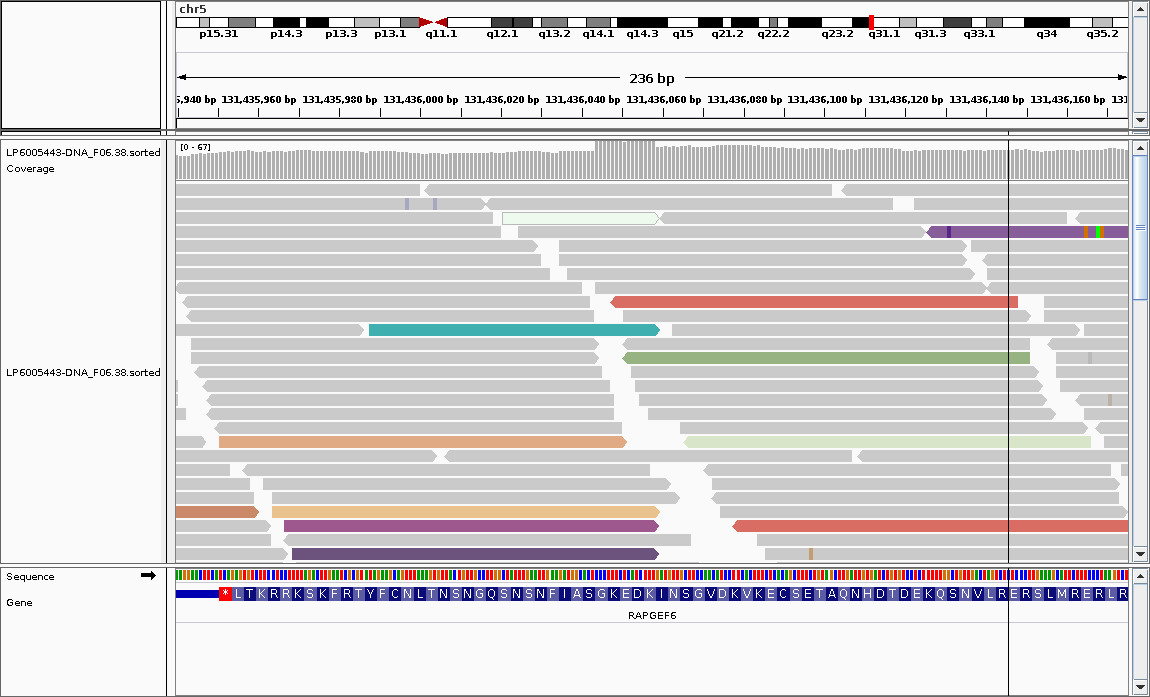


LP6005443-DNA_F06_chr5:131436058

RAPGEF6


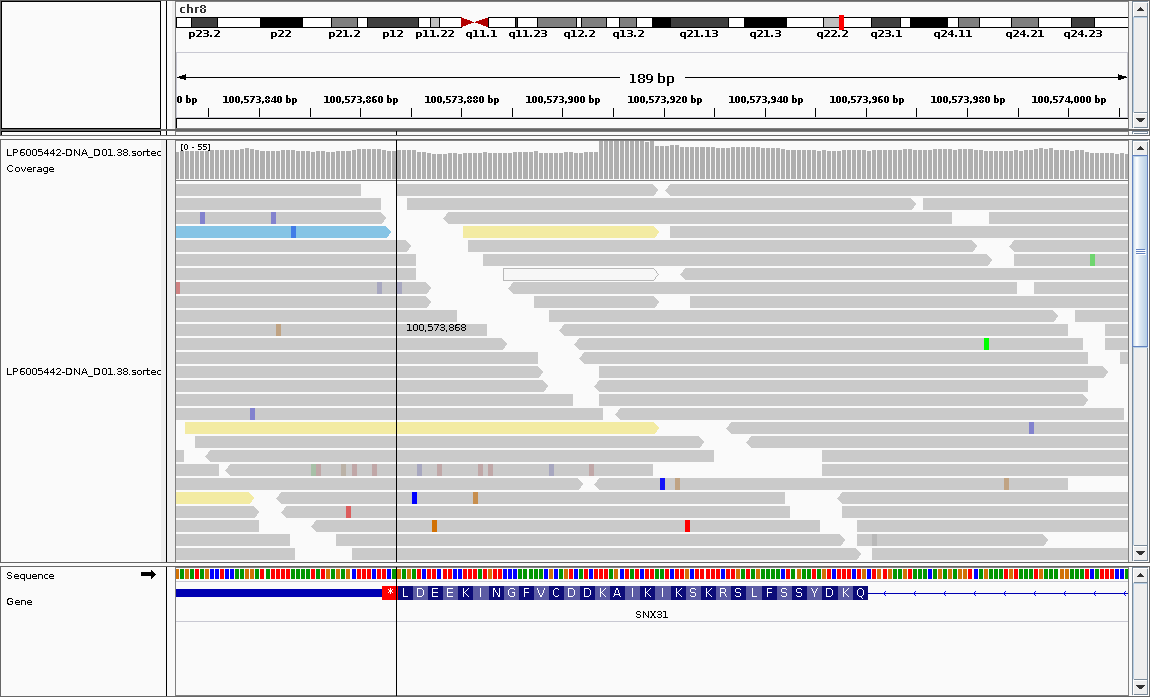


LP6005442-DNA_D01_chr8/100573918

SNX31


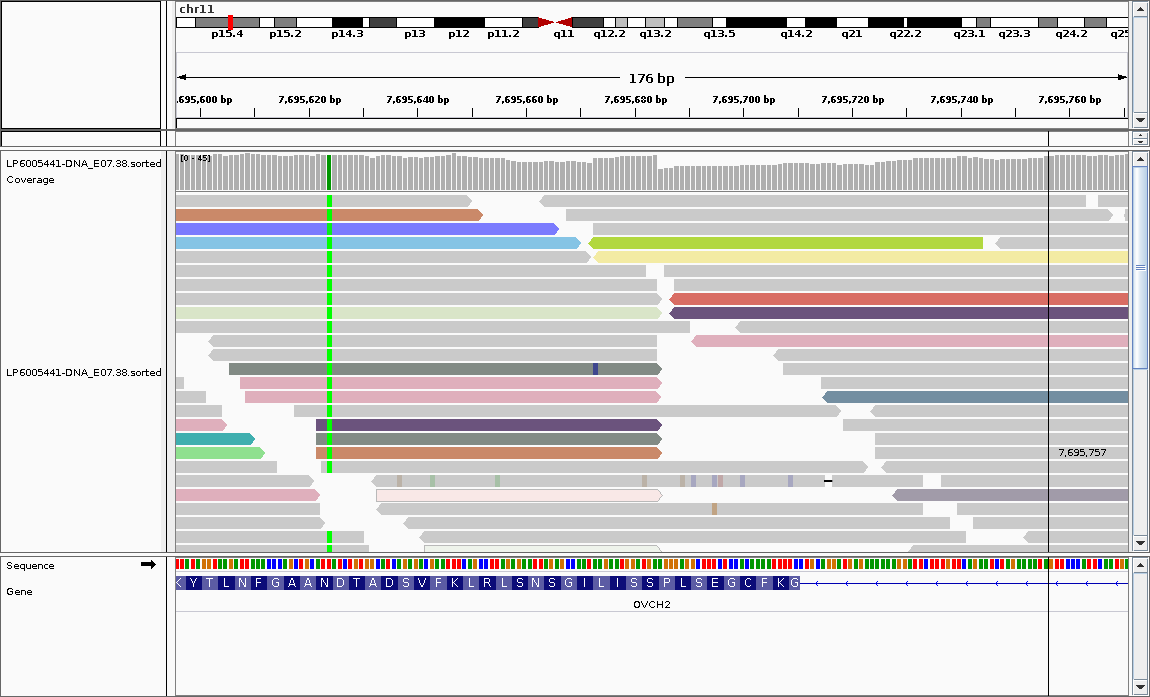


LP6005441-DNA_E07_chr11:7695684

OVCH2


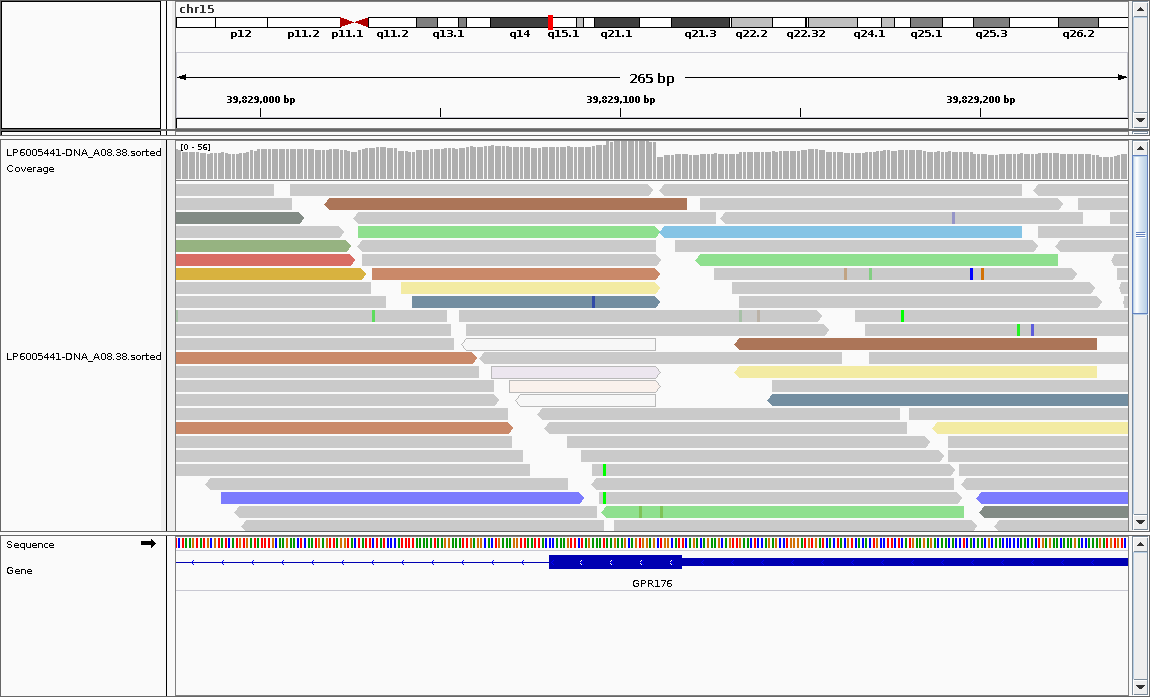


LP6005441-DNA_A08_chr15:39829110

GPR176


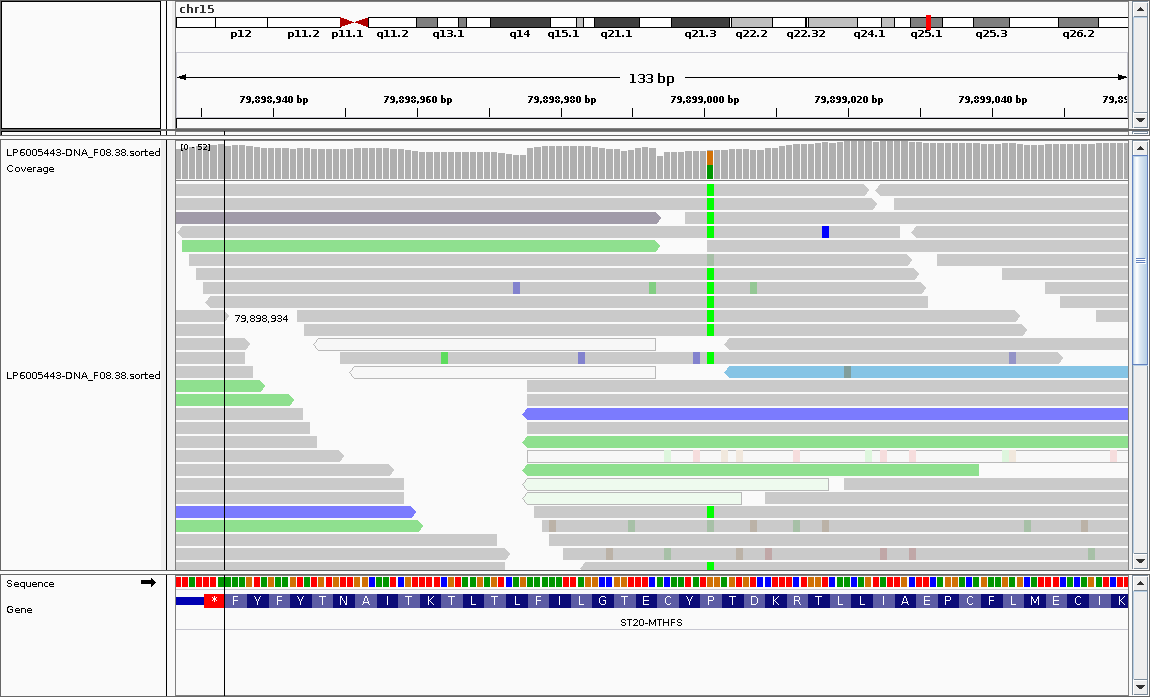


LP6005443-DNA_F08_chr15:79898993

ST20


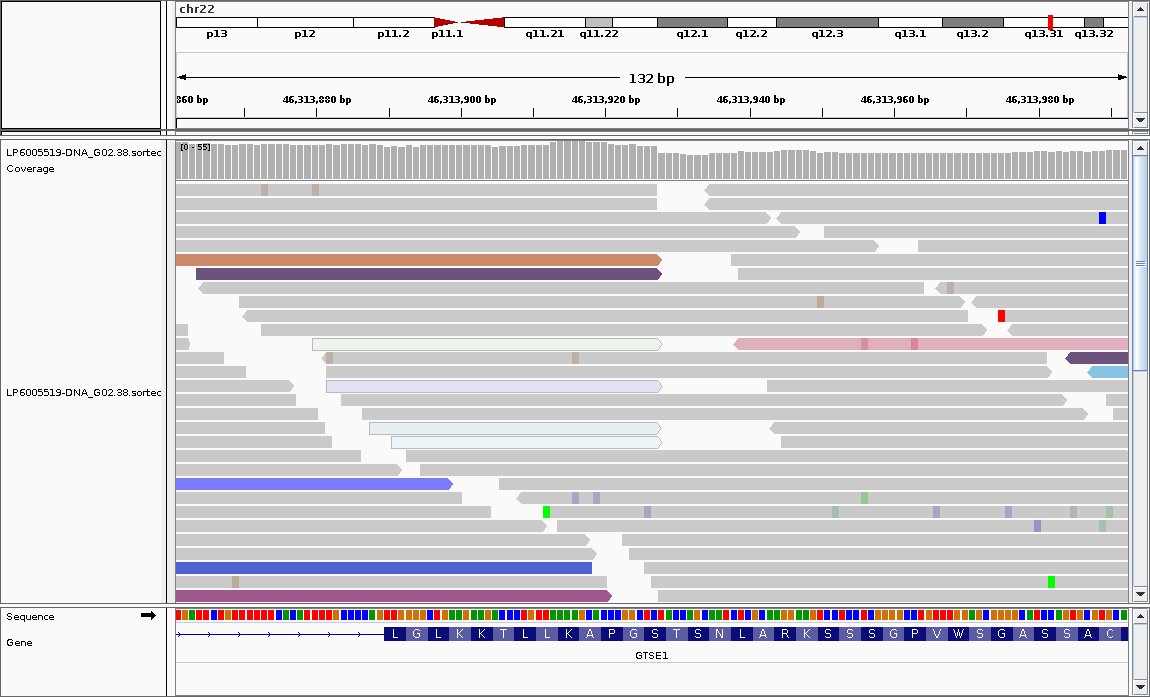


LP6005519-DNA_G02_chr22:46313927

GTSE1

**Supplementary Figure 3. Mobile element insertions disrupt protein coding regions.** Ten *Alu* non-reference retroposon insertions from **Supplementary Table 3** are shown in the Integrated Genome Viewer (IGV) views. Each insertion disrupts the predicted protein product. Each heterozygous MEI insertion event shows the characteristic pattern of normal and discordant reads and higher coverage depth at the target site duplication.

**Supplementary Figure 4. Reference and non-reference MEI neighbor-joining networks for seven major world populations groups.** Neighbor-joining networks depict the genetic distances (Nei’s standard distance) among seven major world population groups. **(a – c)** Population networks based on 11,661 polymorphic non-reference *Alu*, 1,886 non-reference LINE-1 elements, and 475 non-reference SVA elements. **(d – f)** Population networks based on 862 polymorphic reference *Alu* insertion, 94 polymorphic LINE-1 insertions, or 34 polymorphic SVA insertions. Long branch lengths for Africans reflect higher genetic diversity and a greater number of unique mobile element insertions in Africa. Native Americans and Oceanians also display longer branch lengths than other Eurasian groups, which are likely due to bottlenecks and genetic drift in populations with a small effective population size. The greatest genetic (and geographic) distance is consistently between Africans and Native Americans. Native Americans and Central Asians are consistantly clustered. Bootstrap values are shown for the branch points (1000 bootstraps).

| **Supplementary Table 4.  Expected heterozygosity for major population groups** | | | | | | | | | | |
| --- | --- | --- | --- | --- | --- | --- | --- | --- | --- | --- |
|  | **Reference MEIs** | | | **Non-reference MEIs** | | | **All MEIs** | | | **SNPs** |
|  | ***Alu*** | **LINE** | **SVA** | ***Alu*** | **LINE** | **SVA** | ***Alu*** | **LINE** | **SVA** |  |
| **Africans** | 0.378 | 0.280 | 0.315 | 0.077 | 0.052 | 0.044 | 0.097 | 0.063 | 0.062 | 0.278 |
| **South Asians** | 0.345 | 0.248 | 0.332 | 0.052 | 0.039 | 0.025 | 0.072 | 0.049 | 0.046 | 0.269 |
| **West Eurasians** | 0.337 | 0.243 | 0.318 | 0.052 | 0.037 | 0.024 | 0.071 | 0.047 | 0.044 | 0.264 |
| **Central Asians** | 0.337 | 0.254 | 0.295 | 0.049 | 0.036 | 0.022 | 0.069 | 0.047 | 0.040 | 0.255 |
| **East Asians** | 0.322 | 0.244 | 0.275 | 0.048 | 0.038 | 0.024 | 0.067 | 0.048 | 0.041 | 0.251 |
| **Oceania** | 0.315 | 0.229 | 0.302 | 0.048 | 0.038 | 0.023 | 0.066 | 0.047 | 0.042 | 0.239 |
| **New World** | 0.310 | 0.220 | 0.271 | 0.043 | 0.032 | 0.021 | 0.062 | 0.041 | 0.037 | 0.229 |

| **Supplementary Table 5a. Pairwise F_ST_ estimates among populations for *Alu* and LINE-1 MEIs** | | | | | | | |
| --- | --- | --- | --- | --- | --- | --- | --- |
|  | Africans | Americans | Central Asians/  Siberians | East Asians | Oceanians | South Asians | West Eurasians |
| Africans | — | 0.135 | 0.094 | 0.108 | 0.120 | 0.081 | 0.090 |
| Americans | 0.124 | — | 0.047 | 0.067 | 0.130 | 0.079 | 0.095 |
| Central Asians/Siberians | 0.091 | 0.053 | — | 0.012 | 0.079 | 0.030 | 0.047 |
| East Asians | 0.104 | 0.072 | 0.016 | — | 0.070 | 0.036 | 0.065 |
| Oceanians | 0.109 | 0.124 | 0.071 | 0.066 | — | 0.061 | 0.093 |
| South Asians | 0.078 | 0.073 | 0.026 | 0.033 | 0.061 | — | 0.012 |
| West Eurasians | 0.084 | 0.098 | 0.049 | 0.070 | 0.091 | 0.022 | — |
| *Alu* – upper triangle; LINE-1 – lower triangle | | | | | | | |

| **Supplementary Table 5b. Pairwise F_ST_ estimates among world populations for SVA MEIs** | | | | | | | |
| --- | --- | --- | --- | --- | --- | --- | --- |
|  | Africans | Americans | Central Asians/  Siberians | East Asians | Oceanians | South Asians | West Eurasians |
| Africans | — |  |  |  |  |  |  |
| Americans | 0.090 | — |  |  |  |  |  |
| Central Asians/Siberians | 0.050 | 0.062 | — |  |  |  |  |
| East Asians | 0.050 | 0.080 | 0.026 | — |  |  |  |
| Oceanians | 0.066 | 0.123 | 0.071 | 0.043 | — |  |  |
| South Asians | 0.042 | 0.069 | 0.015 | 0.020 | 0.044 | — |  |
| West Eurasians | 0.058 | 0.072 | 0.027 | 0.049 | 0.071 | 0.016 | — |

| **Supplementary Table 6a. Polymorphic *Alu* subfamily proportions by major population** | | | | | | | | |
| --- | --- | --- | --- | --- | --- | --- | --- | --- |
| **MEI sub-family** | **All** | **Africans** | **Americans** | **Central Asians/ Siberians** | **East Asians** | **Oceanians** | **South Asians** | **West**  **Eurasians** |
| *Alu*J/S | 0.0122 | 0.0131 | 0.0192 | 0.0181 | 0.0169 | 0.0192 | 0.0176 | 0.0158 |
| *Alu*Y | 0.0556 | 0.0577 | 0.0669 | 0.0671 | 0.0658 | 0.0611 | 0.0629 | 0.0602 |
| *Alu*Ya/Ya1-4 | 0.2246 | 0.2171 | 0.2171 | 0.2155 | 0.2204 | 0.2017 | 0.2139 | 0.2218 |
| *Alu*Ya5 | 0.2579 | 0.2621 | 0.2487 | 0.255 | 0.2591 | 0.2681 | 0.2556 | 0.2468 |
| *Alu*Yb-non8/6 | 0.0966 | 0.0957 | 0.0971 | 0.0951 | 0.0938 | 0.0882 | 0.0951 | 0.0988 |
| *Alu*Yb8/*Alu*Yb6 | 0.2005 | 0.1984 | 0.1828 | 0.1853 | 0.1878 | 0.1934 | 0.1971 | 0.1983 |
| *Alu*Yc | 0.0772 | 0.0767 | 0.0811 | 0.0781 | 0.071 | 0.0841 | 0.077 | 0.0753 |
| *Alu*Ye-k | 0.0755 | 0.0792 | 0.087 | 0.0859 | 0.0853 | 0.0841 | 0.0808 | 0.0829 |
| Polymorphic loci | 11661 | 7813 | 2183 | 2714 | 3072 | 2652 | 3638 | 4039 |
| Loci/person | 39.4 | 159.4 | 84.0 | 104.4 | 66.8 | 106.1 | 74.2 | 53.9 |

| **Supplementary Table 6b. Polymorphic LINE-1 subfamily proportions by population** | | | | | | | | | | | | | |
| --- | --- | --- | --- | --- | --- | --- | --- | --- | --- | --- | --- | --- | --- |
| **MEI sub-family** | | **All** | | **Africans** | **Americans** | **Central Asians/ Siberians** | **East Asians** | | **Oceanians** | | **South Asians** | **West Eurasians** | |
| L1Ta | | 0.3754 | | 0.3931 | 0.3495 | 0.3418 | 0.3788 | | 0.3579 | | 0.3692 | 0.3472 | |
| L1Ambig | | 0.4364 | | 0.4298 | 0.4637 | 0.481 | 0.481 | | 0.4921 | | 0.4588 | 0.4698 | |
| L1Ta1d | | 0.1856 | | 0.174 | 0.1765 | 0.1696 | 0.1363 | | 0.1474 | | 0.1685 | 0.1762 | |
| LINE-1 | | 0.0027 | | 0.0031 | 0.0104 | 0.0076 | 0.004 | | 0.0026 | | 0.0036 | 0.0069 | |
| Polymorphic loci | | 1886 | | 954 | 289 | 395 | 499 | | 380 | | 558 | 579 | |
| Loci/person | | 6.4 | | 19.5 | 11.1 | 15.2 | 10.8 | | 15.2 | | 11.4 | 7.7 | |
|  |  | |  | |  |  | |  | |  | |  |  |

**Supplementary Table 6c. Polymorphic SVA subfamily proportions by population**

| **MEI sub-family** | **All** | **Africans** | **Americans** | **Central Asians/ Siberians** | **East Asians** | **Oceanians** | **South Asians** | **West Eurasians** |
| --- | --- | --- | --- | --- | --- | --- | --- | --- |
| SVA | 1 | 1 | 1 | 1 | 1 | 1 | 1 | 1 |
| Polymorphic loci | 475 | 225 | 60 | 74 | 115 | 80 | 124 | 142 |
| Loci/person | 1.6 | 4.6 | 2.3 | 2.8 | 2.5 | 3.2 | 2.5 | 1.9 |

| **Supplementary Table 7. *Alu* insertion frequency by subfamily and population group** | | | | | | | | | | |
| --- | --- | --- | --- | --- | --- | --- | --- | --- | --- | --- |
| **Subfamily** | **ALL** | **AF** | **AM** | **CAS** | **EA** | **O** | **SA** | **WE** | **Populations** |  |
| *Alu*Jo | 0.0015 | 0.0017 | 0.0010 | 0.0011 | 0.0016 | 0.0024 | 0.0018 | 0.0012 | All |  |
| *Alu*Sc | 0.0001 | 0.0001 | - | - | 0.0001 | 0.0001 | 0.0001 | 0.0001 | Not CAS, AM |  |
| AluSg | 0.0021 | 0.0022 | 0.0023 | 0.0026 | 0.0019 | 0.0022 | 0.0018 | 0.0019 | All |  |
| *Alu*Sp | 0.0009 | 0.0008 | 0.0014 | 0.0008 | 0.0007 | 0.0007 | 0.0009 | 0.0010 | All |  |
| *Alu*Sz | 0.0120 | 0.0103 | 0.0130 | 0.0127 | 0.0122 | 0.0132 | 0.0122 | 0.0122 | All |  |
| *Alu*Y | 0.0584 | 0.0610 | 0.0547 | 0.0575 | 0.0578 | 0.0535 | 0.0597 | 0.0591 | All |  |
| *Alu*Ya | 0.0672 | 0.0644 | 0.0676 | 0.0680 | 0.0687 | 0.0663 | 0.0675 | 0.0682 | All |  |
| *Alu*Ya1_1 | 0.0264 | 0.0248 | 0.0257 | 0.0258 | 0.0258 | 0.0300 | 0.0276 | 0.0266 | All |  |
| *Alu*Ya1_2 | 0.0067 | 0.0063 | 0.0087 | 0.0074 | 0.0071 | 0.0059 | 0.0067 | 0.0063 | All |  |
| *Alu*Ya2 | 0.0005 | 0.0008 | 0.0002 | 0.0002 | 0.0004 | 0.0004 | 0.0006 | 0.0003 | All |  |
| *Alu*Ya3_1 | 0.0027 | 0.0021 | 0.0031 | 0.0039 | 0.0032 | 0.0029 | 0.0024 | 0.0026 | All |  |
| *Alu*Ya3_3 | 0.0047 | 0.0042 | 0.0045 | 0.0055 | 0.0053 | 0.0045 | 0.0043 | 0.0051 | All |  |
| *Alu*Ya3_4 | 0.0092 | 0.0087 | 0.0098 | 0.0083 | 0.0083 | 0.0077 | 0.0099 | 0.0105 | All |  |
| *Alu*Ya3_5 | 0.0021 | 0.0018 | 0.0024 | 0.0023 | 0.0028 | 0.0015 | 0.0022 | 0.0019 | All |  |
| *Alu*Ya4_1 | 0.0019 | 0.0011 | 0.0021 | 0.0014 | 0.0021 | 0.0023 | 0.0023 | 0.0021 | All |  |
| *Alu*Ya4_2 | 0.0286 | 0.0276 | 0.0298 | 0.0316 | 0.0300 | 0.0289 | 0.0268 | 0.0282 | All |  |
| *Alu*Ya4_3 | 0.0065 | 0.0078 | 0.0040 | 0.0062 | 0.0063 | 0.0069 | 0.0059 | 0.0067 | All |  |
| *Alu*Ya4_4 | 0.0170 | 0.0182 | 0.0155 | 0.0159 | 0.0161 | 0.0200 | 0.0170 | 0.0164 | All |  |
| *Alu*Ya4_5 | 0.0333 | 0.0351 | 0.0306 | 0.0329 | 0.0344 | 0.0330 | 0.0326 | 0.0329 | All |  |
| *Alu*Ya4b | 0.0008 | 0.0005 | 0.0011 | 0.0010 | 0.0008 | 0.0007 | 0.0007 | 0.0010 | All |  |
| *Alu*Ya5 | 0.2442 | 0.2378 | 0.2646 | 0.2490 | 0.2497 | 0.2424 | 0.2416 | 0.2399 | All |  |
| *Alu*Ya5a1 | 0.0031 | 0.0033 | 0.0020 | 0.0035 | 0.0030 | 0.0047 | 0.0024 | 0.0032 | All |  |
| *Alu*Ya5a2 | 0.0082 | 0.0094 | 0.0086 | 0.0078 | 0.0079 | 0.0088 | 0.0081 | 0.0071 | All |  |
| *Alu*Ya5b1 | 0.0097 | 0.0076 | 0.0086 | 0.0100 | 0.0110 | 0.0099 | 0.0108 | 0.0100 | All |  |
| *Alu*Ya5b2 | 0.0001 | 0.0005 | 0.0045 | 0.0001 | - | 0.0001 | 0.0001 | 0.0001 | not EA |  |
| *Alu*Ya5c1 | 0.0038 | 0.0050 | - | 0.0033 | 0.0034 | 0.0034 | 0.0039 | 0.0029 | not AM |  |
| *Alu*Yb | 0.0385 | 0.0410 | 0.0366 | 0.0366 | 0.0382 | 0.0348 | 0.0390 | 0.0389 | All |  |
| *Alu*Yb10 | 0.0009 | 0.0007 | 0.0009 | 0.0007 | 0.0004 | 0.0008 | 0.0012 | 0.0015 | All |  |
| *Alu*Yb11 | 0.0041 | 0.0035 | 0.0032 | 0.0042 | 0.0039 | 0.0054 | 0.0042 | 0.0044 | All |  |
| *Alu*Yb3a1 | 0.0121 | 0.0106 | 0.0115 | 0.0134 | 0.0132 | 0.0108 | 0.0128 | 0.0123 | All |  |
| *Alu*Yb3a2 | 0.0001 | 0.0001 | - | - | - | - | - | - | AF only |  |
| *Alu*Yb5 | 0.0077 | 0.0085 | 0.0092 | 0.0080 | 0.0074 | 0.0070 | 0.0070 | 0.0074 | All |  |
| *Alu*Yb6_1 | 0.0005 | 0.0006 | 0.0002 | 0.0004 | 0.0005 | 0.0002 | 0.0005 | 0.0006 | All |  |
| *Alu*Yb6_2 | 0.0917 | 0.0941 | 0.0855 | 0.0896 | 0.0881 | 0.0950 | 0.0927 | 0.0929 | All |  |
| *Alu*Yb7_1 | 0.0004 | 0.0005 | 0.0002 | 0.0004 | 0.0001 | 0.0002 | 0.0004 | 0.0005 | All |  |
| *Alu*Yb7_2 | 0.0040 | 0.0049 | 0.0043 | 0.0039 | 0.0033 | 0.0032 | 0.0040 | 0.0040 | All |  |
| *Alu*Yb7_3 | 0.0030 | 0.0032 | 0.0031 | 0.0025 | 0.0024 | 0.0038 | 0.0031 | 0.0030 | All |  |
| *Alu*Yb7_4 | 0.0050 | 0.0047 | 0.0038 | 0.0054 | 0.0058 | 0.0043 | 0.0050 | 0.0054 | All |  |
| *Alu*Yb8 | 0.0967 | 0.1023 | 0.0987 | 0.0921 | 0.0924 | 0.0957 | 0.0966 | 0.0959 | All |  |
| *Alu*Yb9 | 0.0077 | 0.0092 | 0.0062 | 0.0071 | 0.0073 | 0.0057 | 0.0076 | 0.0081 | All |  |
| *Alu*Yc | 0.0007 | 0.0004 | 0.0013 | 0.0008 | 0.0003 | 0.0008 | 0.0007 | 0.0010 | All |  |
| *Alu*Yc1 | 0.0706 | 0.0757 | 0.0688 | 0.0707 | 0.0669 | 0.0735 | 0.0698 | 0.0690 | All |  |
| *Alu*Yc2 | 0.0061 | 0.0066 | 0.0053 | 0.0063 | 0.0069 | 0.0060 | 0.0055 | 0.0060 | All |  |
| *Alu*Yd | 0.0002 | 0.0003 | - | 0.0002 | 0.0006 | 0.0003 | 0.0001 | 0.0001 | not AM |  |
| *Alu*Ye | 0.0382 | 0.0359 | 0.0387 | 0.0391 | 0.0399 | 0.0393 | 0.0376 | 0.0385 | All |  |
| *Alu*Ye4 | 0.0022 | 0.0028 | 0.0015 | 0.0016 | 0.0025 | 0.0018 | 0.0023 | 0.0019 | All |  |
| *Alu*Yf | 0.0001 | 0.0001 | 0.0015 | 0.0001 | - | - | - | - | AF, AM, CAS |  |
| *Alu*Yf1 | 0.0018 | 0.0026 | - | 0.0018 | 0.0021 | 0.0021 | 0.0013 | 0.0014 | not AM |  |
| *Alu*Yg | 0.0120 | 0.0101 | 0.0116 | 0.0116 | 0.0132 | 0.0111 | 0.0123 | 0.0131 | All |  |
| *Alu*Yg5b3 | 0.0034 | 0.0032 | 0.0026 | 0.0027 | 0.0033 | 0.0036 | 0.0035 | 0.0041 | All |  |
| *Alu*Yg6 | 0.0138 | 0.0109 | 0.0150 | 0.0141 | 0.0145 | 0.0147 | 0.0153 | 0.0140 | All |  |
| *Alu*Yg6a2 | 0.0045 | 0.0042 | 0.0053 | 0.0055 | 0.0047 | 0.0045 | 0.0046 | 0.0037 | All |  |
| *Alu*Yh | 0.0018 | 0.0009 | 0.0009 | 0.0019 | 0.0017 | 0.0028 | 0.0023 | 0.0023 | All |  |
| *Alu*Yh3 | 0.0002 | 0.0003 | 0.0003 | - | 0.0001 | 0.0005 | 0.0001 | 0.0001 | not CAS |  |
| *Alu*Yh7 | 0.0027 | 0.0030 | 0.0010 | 0.0022 | 0.0020 | 0.0037 | 0.0027 | 0.0032 | All |  |
| *Alu*Yh9 | 0.0001 | - | - | - | - | - | - | 0.0001 | WE only |  |
| *Alu*Yi | 0.0136 | 0.0126 | 0.0140 | 0.0140 | 0.0135 | 0.0132 | 0.0135 | 0.0143 | All |  |
| *Alu*Yi6 | 0.0001 | - | - | 0.0002 | - | - | - | - | CAS only |  |
| *Alu*Yj | 0.0007 | 0.0003 | 0.0006 | 0.0008 | 0.0010 | 0.0003 | 0.0007 | 0.0009 | All |  |
| *Alu*Yj3 | 0.0001 | 0.0001 | - | 0.0001 | - | - | 0.0001 | 0.0001 | AF, CAS, O, WE |  |
| *Alu*Yj4 | 0.0009 | 0.0008 | 0.0013 | 0.0007 | 0.0007 | 0.0002 | 0.0013 | 0.0012 | All |  |
| *Alu*Yk | 0.0002 | 0.0005 | 0.0001 | - | 0.0002 | - | 0.0001 | 0.0002 | not CAS, O |  |
| *Alu*Yk13 | 0.0023 | 0.0015 | 0.0014 | 0.0026 | 0.0027 | 0.0023 | 0.0025 | 0.0027 | All |  |
| Total | 1 | 1 | 1 | 1 | 1 | 1 | 1 | 1 |  |  |
| Abbreviations: AF - Africans; AM - North Americans; CAS - Central Asians and Siberians; EA - East Asians; O - Oceanians; SA - South Asians; WE - Western Eurasians; 63 total subfamilies. Values <0.0001 were rounded to 0.0001. | | | | | | | | | |  |
|  | | | | | | | | | | |
